# Genetic structure and insecticide resistance characteristics of fall armyworm populations invading China

**DOI:** 10.1111/1755-0998.13219

**Published:** 2020-07-20

**Authors:** Lei Zhang, Bo Liu, Weigang Zheng, Conghui Liu, Dandan Zhang, Shengyuan Zhao, Zaiyuan Li, Pengjun Xu, Kenneth Wilson, Amy Withers, Christopher M. Jones, Judith A. Smith, Gilson Chipabika, Donald L. Kachigamba, Kiwoong Nam, Emmanuelle d’Alençon, Bei Liu, Xinyue Liang, Minghui Jin, Chao Wu, Swapan Chakrabarty, Xianming Yang, Yuying Jiang, Jie Liu, Xiaolin Liu, Weipeng Quan, Guirong Wang, Wei Fan, Wanqiang Qian, Kongming Wu, Yutao Xiao

**Affiliations:** ^1^ Shenzhen Branch Guangdong Laboratory for Lingnan Modern Agriculture Genome Analysis Laboratory of the Ministry of Agriculture Agricultural Genomics Institute at Shenzhen Chinese Academy of Agricultural Sciences Shenzhen China; ^2^ State Key Laboratory for Biology of Plant Diseases and Insect Pests Institute of Plant Protection Chinese Academy of Agricultural Sciences Beijing China; ^3^ Tobacco Research Institute of Chinese Academy of Agricultural Sciences Qingdao China; ^4^ Lancaster Environment Centre Lancaster University Lancaster UK; ^5^ Malawi‐Liverpool‐Wellcome Trust Clinical Research Programme Blantyre Malawi; ^6^ School of Forensic and Applied Sciences University of Central Lancashire Preston UK; ^7^ Zambia Agriculture Research Institute (ZARI) Lusaka Zambia; ^8^ Department of Agricultural Research Services (DARS) Bvumbwe Research Station Limbe Malawi; ^9^ DGIMI Univ. Montpellier, INRA Montpellier France; ^10^ National Agro‐Tech Extension and Service Center Beijing China; ^11^ Novogene Bioinformatics Institute Beijing China; ^12^ Grandomics Biosciences, Co., Ltd Beijing China

**Keywords:** gene insertion, population structure, resistance risk, *Spodoptera frugiperda*, subpopulations

## Abstract

The rapid wide‐scale spread of fall armyworm (*Spodoptera frugiperda*) has caused serious crop losses globally. However, differences in the genetic background of subpopulations and the mechanisms of rapid adaptation behind the invasion are still not well understood. Here we report the assembly of a 390.38‐Mb chromosome‐level genome of fall armyworm derived from south‐central Africa using Pacific Bioscience (PacBio) and Hi‐C sequencing technologies, with scaffold N50 of 12.9 Mb and containing 22,260 annotated protein‐coding genes. Genome‐wide resequencing of 103 samples and strain identification were conducted to reveal the genetic background of fall armyworm populations in China. Analysis of genes related to pesticide‐ and *Bacillus thuringiensis* (Bt) resistance showed that the risk of fall armyworm developing resistance to conventional pesticides is very high. Laboratory bioassay results showed that insects invading China carry resistance to organophosphate and pyrethroid pesticides, but are sensitive to genetically modified maize expressing the Bt toxin Cry1Ab in field experiments. Additionally, two mitochondrial fragments were found to be inserted into the nuclear genome, with the insertion event occurring after the differentiation of the two strains. This study represents a valuable advance toward improving management strategies for fall armyworm.

## 1 INTRODUCTION

The fall armyworm, *Spodoptera frugiperda* (J.E. Smith), is a polyphagous pest that is native to tropical and subtropical America, with a strong capacity for migration and reproduction (Johnson, 1987; Mitchell et al., 1991; Westbrook, Nagoshi, Meagher, Fleischer, & Jairam, 2016). It was first detected in Africa in 2016 (Goergen, Kumar, Sankung, Togola, & Tamò, 2016) and spread to 44 African countries within 2 years. It was detected in India in 2018, and has now spread to several southeastern Asian countries (Nagoshi et al., 2020). Such rapid spread poses a global threat to food production. The strong environmental adaptability of fall armyworm is not only reflected in its polyphagy for a wide range of host plants (Luginbill, 1928), but also in its evolution of resistance to chemical pesticides and genetically modified crops expressing *Bacillus thuringiensis* (Bt) toxins (Bernardi et al., 2015; Leibee & Capinera, 1995; Monnerat et al., 2015; Signorini et al., 2018; Storer et al., 2010). Studies have shown that gene families related to detoxification and metabolic processes in the fall armyworm have clearly expanded (Gouin et al., 2017; Liu et al., 2019). In addition, there are two morphologically identical, but genetically distinct, subpopulations or strains of fall armyworm, the rice‐strain (R‐strain) and the corn‐strain (C‐strain), which differ in their host plant selection and sex pheromone composition (Lima & McNeil, 2009; Pashley, 1986; Pashley, Hammond, & Hardy, 1992; Pashley & Martin, 1987). However, there is no absolute mating barrier between the two strains and productive hybridization has been confirmed in both laboratory and field studies (Dumas et al., 2015; Nagoshi, Meagher, Nuessly, & Hall, 2006).

To date, several field‐evolved resistant populations of fall armyworm have been detected, including those displaying resistance to a variety of chemical pesticides and Bt crops (Chandrasena et al., 2018; Gutiérrez‐Moreno et al., 2019; Zhu et al., 2015). The reported mechanisms of resistance to pesticides are mainly due to variation in receptor genes, such as amino acid changes in the ryanodine receptor (RyR) (diamide), acetylcholinesterase (AChE) (organophosphate) and voltage‐gated sodium channel (VGSC) (pyrethroids) (Boaventura et al., 2020; Carvalho, Omoto, Field, Williamson, & Bass, 2013; Yu, Nguyen, & Abo‐Elghar, 2003). In addition, the frame‐shift mutation resulting in early termination of the ATP‐dependent Binding Cassette subfamily C2 gene (*ABCC2*) gene, caused by a 2‐bp insertion, is linked to resistance to Bt toxin Cry1Fa (Banerjee et al., 2017). Field‐evolved strains resistant to Bt toxin Vip3Aa20 were obtained by screening homozygous resistance loci in F_2_ generations in the laboratory (Yang et al., 2018). Clarifying the development of pesticide‐ and Bt‐resistance in fall armyworm would be helpful in providing scientific support for the commercialization of genetically modified crops and Bt biopesticides.

Recent studies have indicated that molecular identification of the C‐ and R‐strains of fall armyworm is dependent on which markers are used (Meagher & Gallo‐Meagher, 2003; Nagoshi, 2012). Early molecular markers based on mitochondrial *Cytochrome Oxidase Subunit I* (*COI*) and Z‐chromosome‐linked *Tpi* genes failed to accurately assign the strain genetic background (Juárez et al., 2014; Nagoshi, 2019; Nagoshi, Goergen, Goergen, Du Plessis, van den Berg, & Meagher, 2019; Nagoshi et al., 2017). The dominant populations of fall armyworm invading Africa and Asia were speculated to be hybrid populations based on these two molecular markers (Zhang et al., 2019). In addition, an Africa‐specific haplotype, different from those native to the Americas, was also reported in African and Chinese samples based on the *Tpi* gene (Liu et al., 2019; Nagoshi et al., 2019), which makes strain identification and studies of population genetic structure more complicated. Therefore, a genome‐wide analysis of the genetic characteristics of invasive fall armyworm is becoming imperative. Although several versions of the fall armyworm genome have now been published (Gouin et al., 2017; Kakumani, Malhotra, Mukherjee, & Bhatnagar, 2014; Liu et al., 2019; Nam et al., 2019; Nandakumar, Ma, & Khan, 2017), a high‐quality genome assembly from a different geographical source is a valuable addition to the genomic resources for this species. Moreover, the different biological properties of the C‐ and R‐strains and the debate regarding strain identification will benefit from further genomic support and explanation. Here we report a chromosome‐level genome sequence of a male moth from an inbred fall armyworm strain, which derived from field populations collected in Zambia in 2017 and would be classed as C‐strain based on *COI* genotype but possessed an Africa‐specific *Tpi* haplotype which differs from the Western Hemisphere (henceforth American) R‐ and C‐strain. We also resequenced 103 fall armyworm samples from 16 Provinces in China, as well as four samples collected from two African countries (Zambia and Malawi). The genome‐wide genetic backgrounds of the invading fall armyworm samples were compared, and insecticide‐resistance risk was assessed based on analysis of potential resistance‐related genes. Comparative genomic analyses of these data will help to reveal the resistance‐related mechanisms and the population genetic characteristics of fall armyworm, which may facilitate its future management.

## 2 MATERIALS AND METHODS

### 2.1 Samples and sequencing for genome assembly

The fall armyworm samples were collected from maize fields in Lusaka, Zambia, in 2017 and reared to produce an inbred strain. One male moth, derived from seven successive generations of single‐pair sib mating, was selected for genomic sequencing for the primary assembly data set and all other individuals used in the Hi‐C and RNAseq experiments were from the same inbred strain. DNA was extracted using the Qiagen Genomic DNA kit (Cat. no. 13323, Qiagen) followed by purity assessment and quantification with a NanoDrop One UV‐Vis spectrophotometer (Thermo Fisher Scientific) and Qubit 3.0 Fluorometer (Invitrogen), respectively. About 0.5 μg genomic DNA (gDNA) was used as input to generate a PCR‐free Illumina genomic library using the Truseq Nano DNA HT Sample preparation Kit (Illumina), with 350‐bp insert size and this library was sequenced in 2 × 150‐bp format on the Illumina NovaSeq 6000 platform. Five micrograms of gDNA from the same individual was used as an input for ~20‐kb insert libraries (SMRTbell Template Prep Kit 1.0, Cat. no. 100‐259‐100, PacBio) sequenced on the PacBio Sequel (Pacific Biosciences). Two third‐instar larvae were selected for Hi‐C library construction, and nuclear DNA was cross‐linked in situ, extracted and then digested with the restriction enzyme *Dpn*II. Hi‐C libraries were amplified by 12–14 cycles of PCR and sequenced on the Illumina NovaSeq 6000 platform with 2 × 150‐bp reads. In addition, three fifth‐instar larvae, three pupae, three female moths and three male moths were used for RNA sequencing. Total RNA was extracted using the RNeasy Mini extraction kit (Qiagen), and a NanoPhotometer spectrophotometer (Implen) and Qubit 2.0 Flurometer (Life Technologies) were used to check the purity and concentration of RNA, respectively. One microgram total RNA per sample was used to make indexed cDNA libraries using the NEBNext Ultra RNA Library Prep Kit for Illumina (NEB) following the manufacturer's recommendations. The libraries had insert sizes of 250–300 bp and were sequenced on the Illumina NovaSeq 6000 platform with 150‐bp paired‐end output.

### 2.2 Genome assembly and correction

The raw PacBio reads longer than 5 kb were assembled into contigs using the software wtdbg2 version 2.4 with the parameters "‐p 0 ‐k 15 ‐AS 2 ‐s 0.05 ‐L 5000" (Ruan & Li, 2019). arrow version 2.1.0 (https://github.com/PacificBiosciences/GenomicConsensus) was used to correct assembly errors after comparing contigs with PacBio reads using pbalign version 0.4.1 (https://github.com/PacificBiosciences/pbalign). The Illumina raw reads were filtered by trimming the adapter and low‐quality regions using clean_adapter version 1.1 with the parameter "‐a Both‐adapter ‐r 75 ‐s 12" and clean_lowqual version 1.0 with the parameter "‐e 0.001 ‐r 75" (https://github.com/fanagislab/ assembly_2ndGeneration/tree/master/clean_illumina). The filtered Illumina reads were aligned to the assembled contigs by bwa mem version 0.7.17 (Li & Durbin, 2009), and single base errors in the contigs were corrected by pilon version 1.21 (Walker et al., 2014).

### 2.3 Genome estimation and evaluation

A distribution analysis of 17 k‐mer frequencies was performed to estimate the genome size of fall armyworm. The filtered Illumina reads were used as input to construct k‐mer frequencies by jellyfish (https://github.com/gmarcais/Jellyfish). Genome size was estimated using *G* = K_num/K_depth, where the K_num is the total number of K‐mers, and K_depth is the frequency occurring more frequently than the others (Li et al., 2010). We used the arthropoda gene set (odb9) to assess the integrity of the genome by Benchmarking Universal Single‐Copy Ortholog (busco) version 3.0.2 (Simao, Waterhouse, Ioannidis, Kriventseva, & Zdobnov, 2015).

### 2.4 Chromsome assembly based on Hi‐C data

The Hi‐C sequencing raw reads were filtered to remove reads containing <5 bases of adaptor sequence; >50% of bases with phred quality value of <19; and <5% of unknown bases (N). Filtered reads were then aligned to the assembled contigs using bowtie2 (version 2.2.3; http://bowtie‐bio.sourceforge.net/bowtie2/index.shtml) (Langmead & Salzberg, 2012). Invalid read pairs were filtered using default settings by hic‐pro (version 2.7.8; https://github.com/nservant/HiC‐Pro) (Servant et al., 2015). lachesis (https://github.com/shendurelab/LACHESIS) (Burton et al., 2013) was applied to cluster, order and orient contigs based on the agglomerative hierarchical clustering algorithm. For each chromosome cluster, the ordered contigs were oriented by building a weighted, directed acyclic graph (WDAG). The orientation of each contig in each chromosomal group was predicted according to the maximum‐likelihood path through WDAG. Finally, we cut the chromosomes predicted by lachesis into bins of equal length (100 kb) and constructed a heatmap based on the interaction signals revealed by valid mapped read pairs between bins using hic‐pro.

### 2.5 Gene prediction and annotation

A de novo repeat library of fall armyworm was constructed by repeatmodeler version 1.0.4 (http://www.repeatmasker.org/RepeatModeler.html). Transposable elements (TEs) were identified by repeatmasker version 4.0.6 (http://www.repeatmasker.org/) using both the de novo library and Repbase library (Repbase‐20150923), and tandem repeats were predicted using tandem repeats finder (Benson, 1999) version 4.07b. We used a combination of ab initio prediction, homology searches and RNA‐seq annotation to predict genes in the *Spodeptera frugiperda* genome. We performed ab initio prediction using augustus 2.5.5 with default parameters (Stanke & Waack, 2003). For homology‐based annotation, we queried the *S. frugiperda* genome sequences against a database containing nonoverlapping protein sequences from closely related species (*Bombyx mori*, *Helicoverpa armigera*, *Spodoptera litura*) by genblasta with default parameters (She, Chu, Wang, Pei, & Chen, 2009). genewise (Birney, Clamp, & Durbin, 2004) was used to refine the genblasta mappings to the genome. For the RNA‐seq annotation, the RNA‐seq data were mapped to the assembled genome of *S. frugiperda* using tophat version 2.0.12 and alignments were processed by cufflinks version 2.2.1 with default parameters to generate transcript predictions (Trapnell et al., 2012). evidence modeler (Haas et al., 2008) version 1.1.1 was used to combine ab initio predictions, homology‐based searches and RNA‐seq alignments. Predicted gene models supported by at least one of the annotations using the UniProt database, NR database and RNA‐seq data were retained. Gene functional annotation was performed by aligning the predicted protein sequences to the NCBI NR, UniProt, eggNOG, and KEGG databases with blastp version 2.3.0+, applying an *E*‐value cut‐off < 10^−5^.

### 2.6 Phylogenetic tree construction and genomic comparison

Orthologous and paralogous gene families identified in a set of 10 species (*Drosophila melanogaster*, *Plutella xylostella*, *Bombyx mori*, *Manduca sexta*, *Danaus plexippus*, *Heliconius melpomene*, *Operophtera brumata*, *Helicoverpa armigera*, *Spodoptera frugiperda*, *Spodoptera litura*) with published genomes were analysed by orthofinder version 2.3.1 with default parameters. Orthologous groups that contain single‐copy genes for each species were selected to construct the phylogenetic tree. The multisequence alignment of proteins was accomplished by muscle (Edgar, 2004) version 3.8.31. A neighbour‐joining (NJ) phylogenetic tree was constructed using mega version 7.0.14. The current assembled genome was aligned with two published versions of fall armyworm genomes using the mummer3.23 (Kurtz et al., 2004) package with cutoff of identity >80% and coverage >80%. Alignments were filtered to generate a multi‐alignment data set using the delta‐filter utility with 85% minimum identity (‐i 85) and minimum alignment length 10 (‐l 10). A set of unique alignments was created using the same filter criteria but with the addition of the ‐r and ‐q flags.

### 2.7 Sampling for resequencing and population genetic study

A total of 103 Chinese fall armyworm samples were used for resequencing. All samples were collected as larvae on maize or sugarcane from 50 cities of 16 provinces (autonomous regions or municipalities) of China. The larvae were fed with fresh maize leaves and brought back to the laboratory under ambient conditions during transportation. Larval bodies were cleaned and then stored in a freezer at −80°C. Detailed sample information is presented in Table S1 and the sample distribution in China is shown in Figure S1. In addition, four fall armyworm samples from Africa were also used for resequencing, including two samples (AFR4 and 5) from the same inbred strain (AFR2017) as the genome sequencing in this study, and another two samples (AFR14 and 15) which were collected from maize fields in Bvumbwe, Malawi, in January 2019, which is also an inbred strain (AFR2019) reared in the laboratory. A total of 1.5 μg gDNA of each sample was used to construct a 350‐bp insert library using the Truseq Nano DNA HT Sample preparation Kit (Illumina) sequenced in 150‐bp paired‐end mode as described in section 2.1. Raw reads were aligned to the NCBI NT database using blastn, and reads with significant matches (identity > 95% and coverage > 80%) to microbes or host plants were removed.

A further 173 fall armyworm samples from 21 provinces in China were used for strain identification and molecular detection using PCR amplification and Sanger sequencing. Genomic DNA was extracted using the Multisource Genomic DNA Miniprep Kit (Axygen) according to product instructions. The 50‐µl PCR mixture contained 25 µl of 2 × Easytaq mix, a mixture of 2 µl forward and reverse primers (10 μM), 2 µl DNA, and 21 µl diethy1 pyrocarbonate (DEPC) H_2_O. PCR was performed at 94°C for 5 min, 34 cycles of 94°C for 30 s, 60°C for 30 s and 72°C for 30 s, and finally 72°C for 5 min. A total of 10 µl of the PCR products containing the target fragment were sequenced by Life Technology. These samples were collected from the field as larvae or adult moths. Detailed sample information is presented in Table S2 and the sample distribution in China is shown in Figure S1. Mitochondrial *COI* and *Tpi* markers were used for strain identification. ABCC2 and AChE genes were detected based on primers designed according to published mutation sites (Banerjee et al., 2017; Carvalho et al., 2013). Inserted mitochondrial fragments in the nuclear genome were detected using primers designed in this study. All primer sequence information in this study is shown in Table S3.

### 2.8 Read mapping and SNP calling

The Illumina raw reads from resequenced samples were filtered using clean_adapter and clean_lowqual software as described in section 2.1, resulting in high‐quality reads with an average error rate of <0.01. The high‐quality reads were then aligned to the fall armyworm reference genome (American C‐strain) and mitochondrial genome sequences using bwa mem software (Li & Durbin, 2009) version 0.7.5a with default parameters. Alignments for each sample were processed by removing duplicate reads using the samtools (Li et al., 2009) software package version 1.3. The mpileup function in samtools was used to generate mpileup files for each sample. vcftools (Li, 2011) was used to identify SNPs and small indels. Several criteria were considered in SNP filtering: (a) a read mapping score higher than 40; (b) minimum coverage greater than 10; and (c) SNP genotypes called in >90% of samples. We also conducted principal component analysis (PCA) to evaluate genetic structure using the software Genome‐wide Complex Trait Analysis (gcta) version 1.04 (Yang, Lee, Goddard, & Visscher, 2011).

### 2.9 Bioassays of insecticides and Bt maize in the field

Bioassays were conducted by a topical application procedure (Armes, Jadhav, Bond, & King, 1992). Two inbred strains (cdcc and cdyc) collected from Yunnan Province and reared for multiple generations in the laboratory were tested using 14 types of pesticide commonly used in agricultural production (Table S4). Drops (1.0 µl) of a serial dilution of technical insecticides in acetone solution were applied with a micropipette to the thoracic dorsum of the third‐instar larvae, with control larvae treated with 1.0 µl acetone. After treatment, the larvae were reared individually in 24‐well plates containing ad libitum artificial diet without any Bt proteins or insecticides. Larvae were retained in an insect chamber with a controlled environment of 26 ± 1°C, 60 ± 10% relative humidity and a photoperiod of 16 hr: 8 hr (light–dark). Mortality was assessed after 72 hr of treatment. Larvae were considered dead if they were unable to move in a coordinated manner when prodded with a small soft brush. We used median lethal doses (LC_50_) to evaluate the resistance level of different fall armyworm populations. The LC_50_ and 95% fiducial limit (FL) for each insecticide were estimated by probit analysis using the software package polo‐pc (Russell, Robertson, & Savin, 1977) (LeOra Software).

The Bt toxin field bioassays were conducted at a genetically modified (GM) test base in Yunnan Province, China. Test seeds of GM maize (expressing Cry1Ab) and control maize were provided by the DBN Biotech Center, Beijing DBN Technology Group Co., Ltd. Both maize types were planted in ~180 m^2^, with each type being replicated three times. Larval density and maize damage rates were investigated at different growth stages of maize at seven different dates during June and July. The investigation was performed in a five‐spot sampling method with 20 maize plants per point. Fall armyworm damage assessment was performed according to standard procedures (Davis, Ng, & Williams, 1992; Williams, Buckley, & Daves, 2006; Wiseman & Widstrom, 1984).

## 3 RESULTS

### 3.1 High‐quality genome assembly of fall armyworm

**FIGURE 1:**
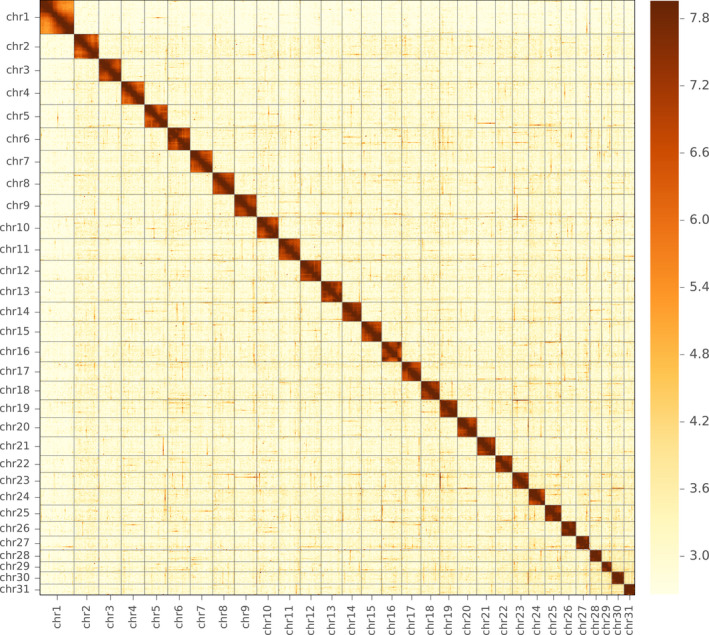
A genome‐wide contact matrix from Hi‐C data between each pair of the 31 chromosomes [Colour figure can be viewed at wileyonlinelibrary.com]

A total of 25.89 Gb raw PacBio long reads and 162.4 Gb Illumina raw reads were generated. After filtering low‐quality and duplicated reads, 24.72 Gb PacBio long reads and 95.4 Gb high‐quality Illumina reads were used for genome assembly, together representing an ~300× coverage of the fall armyworm genome. Using wtdbg2 (Ruan & Li, 2019), the final genome was assembled into 776 contigs with size of 390.38 Mb and contig N50 length of 5.6 Mb (longest, 18.5 Mb), including a complete mitochondrial sequence (Table 1). The assembled genome size was close to the estimated size of 395 Mb based on k‐mer depth distribution analysis, which was also similar to that determined by flow cytometry (396 ± 3 Mb) (Gouin et al., 2017). After interaction analysis based on a total of 78 Gb data obtained through Hi‐C sequencing, 143 contigs were concatenated into 31 linkage groups with a scaffold N50 of 12.9 Mb, accounting for 96.3% of the total genome length (Figure 1). By aligning the Illumina data with the assembled fall armyworm genome, the mapping rate and coverage were 98.8% and 99.7% (≥5 reads) respectively, highlighting the accuracy and high integrity of the genome assembly. The genome size reported in this study is intermediate between those of previously published fall armyworm versions, but the genome is nearly 140 Mb smaller than that recently published by Liu et al. (2019). Genome collinearity analysis showed that more than 98% of the current assembled genome was consistent with previously published versions (Gouin et al., 2017; Liu et al., 2019) (Table S5), and regions within the assembly presented in this study align to multiple regions of Liu's assembly, indicating the previous assembled genome with larger size was mainly caused by high heterozygosity of sequenced samples.

**TABLE 1:** Summary of assembly results of *Spodoptera frugiperda*

Assembly feature	FAW (this study)	FAW (corn strain)	FAW (rice strain)
Assembled sequences (Mb)	390	438	371
Longest scaffold size (kb)	21,916.7	943.2	314.1
N50 size of scaffold (kb)	12,966.7	52.8	28.5
N90 size of scaffold (kb)	7,574.2	3.5	6.4
Longest contig size (kb)	18,555.4	362.9	191.4
N50 size of contig (kb)	5,606.9	16.9	24.3
N90 size of contig (kb)	991.8	2.9	5.6
GC content in genome (%)	36.4	36.0	36.1
Number of gene models	22,260	21,700	26,329
busco complete gene (%)	98.4	88.1	93.5
busco duplicated gene (%)	2	11.3	2
busco missing gene (%)	1.4	4.2	2.3

By combining homology‐based and de novo approaches, we identified ~27.2% of repetitive elements in the assembled fall armyworm genome. Among the known repeat families, long interspersed nuclear elements (LINE) constituted the most abundant repeat family, representing 8.7% of the repetitive sequences, while long terminal repeats (LTR) comprised only 1.4% (Table S6). To annotate the fall armyworm genome, we performed deep transcriptome sequencing of larvae, pupae, and male and female moths, including three different developmental stages, which generated 98.4 Gb of RNA‐seq data. By combining homologue‐based, ab initio and transcriptome‐based approaches, we predicted 22,260 protein‐coding genes (gene models) in the fall armyworm genome, which is greater than the number of predicted genes in other lepidopteran genomes that have so far been published (Dasmahapatra et al., 2012; Kanost et al., 2016; Pearce et al., 2017; Wan et al., 2019; Xia et al., 2008; You et al., 2013; Zhan, Merlin, Boore, & Reppert, 2011). More than 85.5% of the predicted coding sequences (CDS) were supported by transcriptome sequencing data (defined as when ≥ 70% of the predicted CDS of a gene was covered by transcriptome reads). Further assessment of assembly integrity based on busco analysis showed that the current genome contained 98.4% complete BUSCO genes.

**FIGURE 2:**
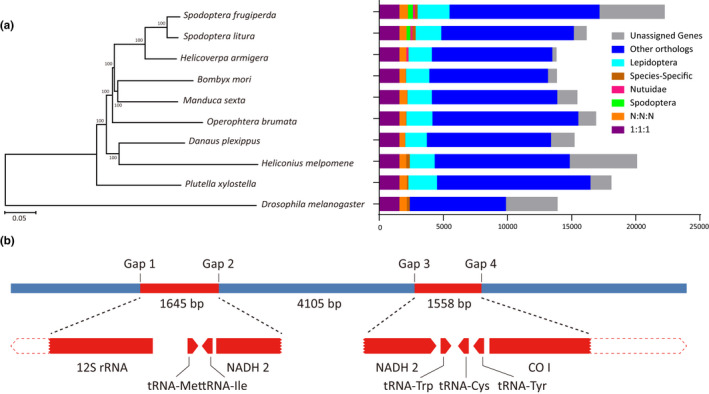
Phylogenetic relationships and schematic map of mitochondrial insertion. (a) Phylogenetic tree and genomic comparison of 10 species of Lepidoptera and Diptera. *Drosophila melanogaster* was used as an outgroup and bootstrap value was set as 1,000, 1:1:1 includes the common orthologues with the same number of copies in different species, N:N:N includes the common orthologues with different copy numbers in different species, other orthologues include the unclassified orthologues, and unassigned genes include the genes that cannot be clustered into known gene families. (b) A schematic map of two mitochondrial fragments inserted into the nuclear genome; the *NADH2* gene was separated by a 4,105‐bp fragment, and both of two inserted mitochondrial fragments were identical with the C‐strain genotype [Colour figure can be viewed at wileyonlinelibrary.com]

Comparative analysis of orthogroups of nine Lepidoptera species and *Drosophila melanogaster* (Diptera) was performed (Table S7). Among them, 17,180 genes in 10,755 orthogroups were found in the current genome of fall armyworm, and the remaining 5,080 lineage‐specific genes were identified as unassigned genes. Compared with *Spodoptera litura*, *S. frugiperda* has more species‐specific genes, and the number of unassigned genes is much greater than that of *S. litura* (Figure 2a). Phylogenomic analyses of the 10 species were conducted using 1,571 single‐copy genes. As shown in Figure 2a, the taxonomic relationship and phylogenetic status of current species was similar to phylogenetic analyses based on 13 mitochondrial protein‐coding genes (Lämmermann, Vogel, & Traut, 2016). Three species of Noctuidae, including *S. frugiperda*, formed one group, which then clustered with *Bombyx mori* (Bombycidae) and *Manduca sexta* (Sphingidae). Two butterflies, *Danaus plexippus* and *Heliconius melpomene* (both Nymphalidae), clustered together as an outer branch, while *Plutella xylostella* (Plutellidae) is the outermost branch of Lepidoptera (Figure 2a).

### 3.2 Genetic background of fall armyworm populations in China

**FIGURE 3:**
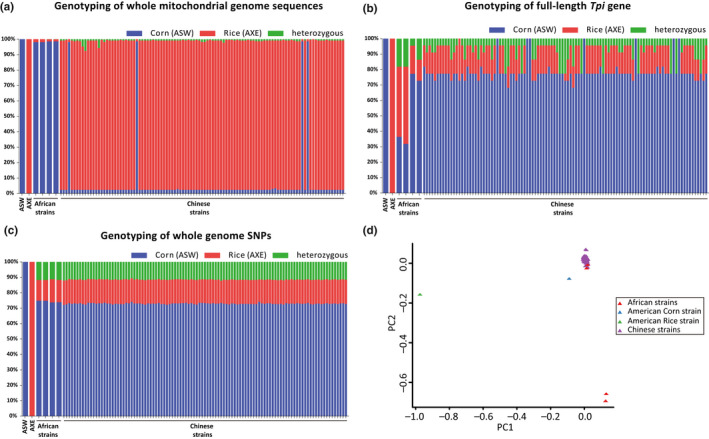
Genetic background of 107 fall armyworm samples. (a) Genotyping based on 208 mitochondrial SNP loci. From left to right, the leftmost two samples are ASW (the American corn strain) and AXE (the American rice strain), four African strains (AFR4 and 5 from Zambia, then AFR14 and 15 from Malawi), and 103 strains from China; the order of each sample is consistent with Table S1. (b) Genotyping based on 22 SNP loci in the *Tpi* gene. (c) Genotyping based on 707,353 genome SNP loci. (d) Principal component analysis (PCA) based on 5,998,089 whole‐genome SNPs. Colour codes indicate samples from different sources; the two samples at the bottom in red are African samples AFR4 and AFR5 [Colour figure can be viewed at wileyonlinelibrary.com]

A total of 103 fall armyworm samples from China were resequenced, as well as four samples from two countries in Africa (Zambia and Malawi). The generated Illumina data ranged from 8.6 to 18.9 Gb for each sample, with a median genome coverage of 32.5×. First, we analysed the whole mitochondrial genome sequences of all samples. A total of 208 SNP loci were selected for analysis, based on comparison of the published mitochondrial sequences of both the American R‐strain (AXE) and C‐strain (ASW) (Gouin et al., 2017). Genotypes were obtained at these 208 sites for each individual after mapping the filtered sequence reads to the assembled mitochondrial genome. We found that most of the samples were assigned to the R‐strain, and all four samples from Africa were C‐strain, while only four out of 103 samples in China were assigned to the C‐strain based on the mitochondrial genome (Figure 3a). Note that most R‐strain samples surprisingly contain heterozygous mitochondrial SNPs, which could be caused by inserted C‐strain fragments or existing standing variation of low frequency. The proportion of the C‐strain in this sample set was ~10% and was similar to that of the 173 Chinese fall armyworm samples identified by PCR based on the *COI* gene in this study (Table S2).

**FIGURE 4:**
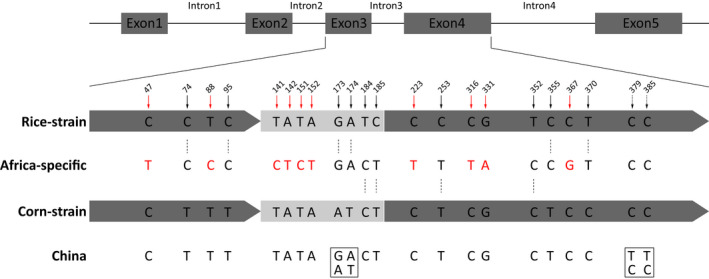
Diagram of the *Tpi* gene segments with respect to consensus Western Hemisphere sequences and the haplotypes observed in samples collected from Africa and China. Black solid arrows indicate 10 SNPs used to identify the American R‐strain and C‐strain fall armyworm, in which P370 was considered to be an effective diagnostic marker. Red solid arrows indicate 10 SNPs specific to the Africa‐specific strain. The boxes represent two variable loci in some Chinese samples, including homozygous or heterozygous genotypes [Colour figure can be viewed at wileyonlinelibrary.com]

Next, we analysed the *Tpi* gene, which is commonly used in strain identification of fall armyworm (Nagoshi, 2012). By comparing the full‐length *Tpi* gene of the American R‐strain (AXE) and C‐strain (ASW), 22 SNP loci were found. The genotype of each individual was analysed based on these 22 sites. The results showed that all fall armyworm samples collected from China contained more C‐strain SNP loci, as did the Malawi samples (AFR14, AFR15), but not those from Zambia samples (AFR4, AFR5) which represents the Africa‐specific haplotype and which contained ~50% of R‐strain SNP loci. Genotypes of seven Chinese samples were identical to the American C‐strain (ASW) and the remaining samples contained a small proportion of R‐strain genotypes or heterozygous SNPs (Figure 3b). However, none of the samples was found to be identical to the American R‐strain genotype (AXE). We further used PCR to analyse genotypes of 173 samples based on 10 strain‐biased SNPs within the *Tpi* gene reported previously (Nagoshi, 2012). The results showed that almost all of the samples correspond to C‐strain genotypes, although three samples (G‐GXW11, G‐GXW13, G‐EP6) were identified as an Africa‐specific haplotype, which was significantly different from known R‐ or C‐strain genotypes (Figure 4; Table S2). In summary, our genotyping results show that there are obvious contradictions between strain identification using mitochondrial and *Tpi* gene markers.

To clarify the genetic background of fall armyworm populations invading China, we screened a total of 707,353 SNPs exhibiting homozygous differences between the reference American R‐strain (AXE) and C‐strain (ASW) in the 107 resequenced samples (Figure 3c). The results showed that all the samples, including the four from Africa, had more than 70% of the genetic background of the American C‐strain (ASW) genotype. The proportion of R‐strain SNPs was about 15%, and the remaining 12% were heterozygous. The results showed that fall armyworm invading China have a dominant percentage of the C‐strain background. PCA based on 5,998,089 whole‐genome SNPs also demonstrated that samples from China were much closer to the C‐strain (ASW) than to the R‐strain (AXE), in which PC1 explained 6.45% of the variation. African samples from Zambia (AFR4, AFR5) were separated on PC2, which explained 2.15% of the variation (Figure 3d). By comparing the results of the mitochondrial genome, *Tpi* gene and genome‐wide identification, it becomes apparent that there is no correlation between the mitochondrial and whole genome genotype. Although *Tpi* genotyping shows results more similar to those of the whole genome, the presence of the Africa‐specific *Tpi* haplotype increases the complexity of using this marker for identification.

### 3.3 Fall armyworm is developing a high risk of resistance to conventional pesticides

Insecticide resistance evolution is one of the most challenging problems in the control of fall armyworm. Identifying resistance‐related genes is helpful for the monitoring and prevention of fall armyworm outbreaks. We selected 14 previously reported resistance‐related genes of lepidopteran pests and scanned the resequenced samples to analyse variation in target genes. The results showed that all the target genes had multiple variation sites with a high frequency of SNPs in the CDS region (Table S8).

**FIGURE 5:**
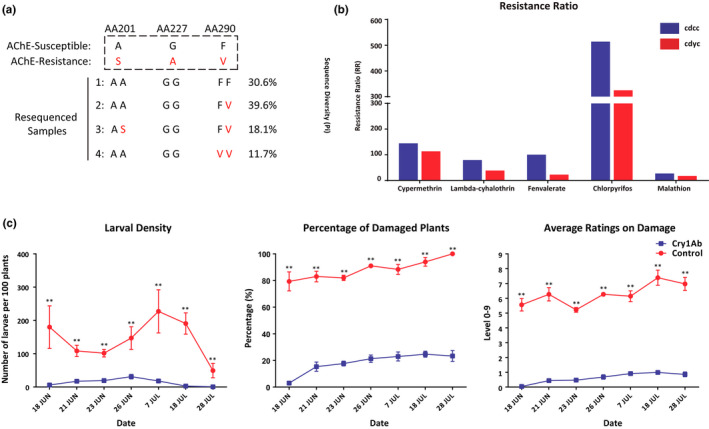
Genome scans and bioassays of fall armyworm for insecticide resistance. (a) Genotype and resistance mutation sites of the AChE gene in fall armyworm populations in China. (b) The resistance ratios (RRs) of two Chinese fall armyworm populations to pyrethroid (cypermethrin, lambda‐cyhalothrin, fenvalerate) and organophosphate (chlorpyrifos, malathion) insecticides; cdcc and cdyc represent two inbred strains collected from Yunnan Province in China. RRs were calculated from the LD_50_ (µg/g) of a field population over the LD_50_ of a susceptible population as in Yu (1991). (c) Resistance tests of GM maize and non‐GM maize to fall armyworm in field experiments. Error bars are the *SD* (*n* = 15), and asterisks indicate significant differences based on Student's *t* test (**p < .01) [Colour figure can be viewed at wileyonlinelibrary.com]

Studies have shown that the amino acid substitutions in AChE (A201S, G227A, F290V), VGSC (T929I, L932F, L1014F) and RyR (I4790M, G4946E) result in resistance to organophosphate, pyrethroid and diamide insecticides, respectively. The results of variation scanning of the 107 resequenced samples showed that resistance mutations were present in amino acids 201 and 290 of AChE (Figure 5a). Among them, the first locus had 17.1% heterozygous mutations, and the third locus had 29.7% homozygous resistance mutations and 58.2% heterozygous mutations. No resistance mutations were detected at the targeted sites of the VGSC and RyR gene in any samples. We also designed primers to detect the resistance mutation sites in *AChE* in 173 Chinese samples by PCR amplification and Sanger sequencing. The results were similar to the Illumina data, showing that ~75% of samples have homozygous or heterozygous variation at amino acid 290.

**FIGURE 6:**
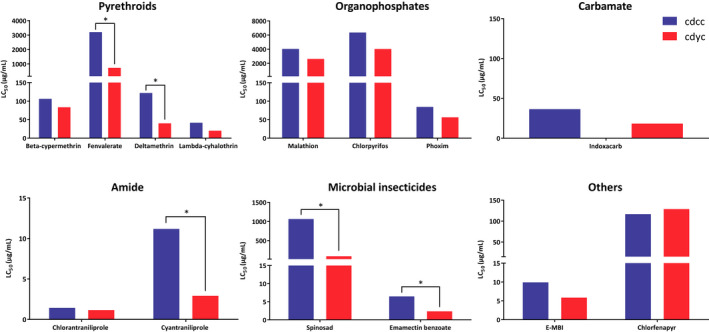
The LC_50_ values of two Chinese fall armyworm populations to different kinds of insecticides. cdcc and cdyc represent two inbred strains collected from Yunnan Province in China. Statistical significance of the difference was assessed by whether the 95% fiducial limits overlap (*p < .05) [Colour figure can be viewed at wileyonlinelibrary.com]

To understand the baseline susceptibility of fall armyworm invading China, we determined the LC_50_ values to 14 insecticides for two Chinese fall armyworm populations collected from Yunnan Province. The results showed that the LC_50_ for both fall armyworm populations to chlorpyrifos, a fenvalerate, were at relatively high level, and well above those of the laboratory‐susceptible *Helicoverpa armigera* strain (Bird & Downes, 2014). The LC_50_ to chlorantraniliprole was low, as were those to emamectin benzoate and indoxacarb, which were similar to results of a previous study on *H. armigera* and could be considered as the susceptible baseline (Bird, 2015) (Figure 6). Resistance levels of the two populations to pyrethroids and organophosphate pesticides were very high; in particular, resistance ratios to chlorpyrifos of the two populations were more than 300‐fold compared to a laboratory susceptible fall armyworm population that was sampled in 1975 (Yu, 1991) (Figure 5b). These results provide a susceptible baseline for fall armyworm populations invading China to different pesticides, which can provide guidance for resistance monitoring and pesticide management strategies.

### 3.4 Fall armyworm invading China are currently sensitive to Bt toxin in a field‐evolved experiment

The insertion of 2 bp in the *ABCC2* gene of fall armyworm was reported to cause a frame‐shift mutation and results in resistance to Cry1Fa (Banerjee et al., 2017). We did not detect the same insertion mutation in 107 resequenced samples nor in 173 samples screened by using PCR and Sanger sequencing. Although the percentage of SNPs in the CDS region of other Bt receptors such as *SR‐C* (scavenger receptor class C gene, a specific receptor for Vip3Aa in Sf9 cells), *TSPAN1* and other ABC gene families related to Cry toxin were also very high (Table S8), no reported resistant mutation was found in any target resistance genes.

Field tests showed that fall armyworm samples invading China were sensitive to GM maize expressing Cry1Ab compared with the control group. Damage assessment on larval density, the percentage of damaged plants and average damage ratings of GM maize were significantly lower than those of the control group (Figure 5c), indicating that the GM maize expressing Cry1Ab currently has good control effects on the invading population of fall armyworm in China.

### 3.5 Insertion of mitochondrial fragments into the nuclear genome in a recent evolution event

We found that two mitochondrial fragments, with sequence lengths of 1.5 kb (partial *COI* gene and *NADH2* gene) and 1.6 kb (partial *NADH*2 gene and 12S rRNA gene), were inserted into the nuclear genome, separated by a 4.1‐kb segment of the nuclear genome (Figure 2b). The total length of a ~ 7.3‐kb fragment, including two inserted fragments, was supported by more than 28 raw reads of PacBio data. The lengths of all 28 reads were longer than 20 kb and completely covered the 7.3‐kb fragment. However, the two insertions were not found in other published fall armyworm genomes. To verify the accuracy of this result, we designed four primers based on flanking sequences of four connection points (Gap1–4 in Figure 2b), and the results of PCR amplification confirmed the existence of the insertion. The same primers were used in PCR assays to detect the insertion in 173 fall armyworm samples and it was found that the insertion was present in only 26.0% of all samples (Table S2). At the same time, the resequencing data of 107 fall armyworm samples in this study also showed that there were varying numbers of reads covering the four junction points in 29 samples, and the percentage of samples with inserted reads was 27.1% (Table S9). Both the PCR and resequencing results showed that the insertion was not present in all samples, perhaps suggesting that it has a recent evolutionary origin.

Moreover, the genotype of the two inserted mitochondrial fragments was identical to that of the C‐strain, indicating that the insertion occurred after differentiation of the R‐ and C‐strains. Further analysis indicated that the two mitochondrial fragments were inserted into the intron region of the lysine‐specific demethylase 3 B (*Kdm3B*) gene, which is not likely to affect expression of the gene. The inserted partial *COI* and *NADH2* gene fragments were also considered likely to be functionless.

## 4 DISCUSSION

The rapid spread of the fall armyworm has attracted popular attention worldwide. Accurate identification of its genetic characteristics (strain and pesticide resistance properties) has a direct and practical importance in terms of risk assessment and control strategies. A genome‐wide analysis can reveal more in‐depth genetic information than conventional gene‐level analyses. The results of this study show that the fall armyworm invading China has a genetic background dominated by American corn‐strain genotypes. Most of the fall armyworm samples invading China were detected and collected from corn and sugarcane, which are more likely to show the characteristics of C‐strain host plants. Along the invasion path of the migratory fall armyworm, there are large‐scale rice planting areas in Southeast Asia and central China, although there are few reports of serious damage to rice caused by fall armyworm (http://www.fao.org/fall‐armyworm). The established R‐strain fall armyworm in the Americas mainly feeds on turf grass, and there were few reports of damage to rice in 1970s (Bowling, 1978; Gallego, 1967). In addition, the established R‐strain *Tpi* genotype has not been detected in any of the samples collected from Africa or Asia. We therefore speculate that the American R‐strain fall armyworm did not invade Africa or Asia, including China.

In our study, 103 resequenced Chinese samples were collected from different regions of 50 cities distributed across 16 provinces (Figure S1). The collection time and sites coincided almost perfectly with the spreading invasion of fall armyworm in China. However, there was no obvious correlation between the time or site of collection and the genetic structure of the fall armyworm population (Figure 3). Almost all samples have similar genomic backgrounds, which suggests that the invading population may originate from a single genetic source and there is no evidence for genomic selection during the invasion.

According to our results, commonly used strain identification of fall armyworms by mitochondrial or *Tpi* markers is limited or even inaccurate. The nuclear insertion of two C‐strain partial *COI* fragments in this study further underlines the need for caution in interpreting mitochondrial genotypes. We also found that the AT/GC SNP located at *Tpi*‐intron3 (P173/174) was inadequate as a diagnostic marker. In addition, the TT/CC SNP located at *Tpi*‐exon4 (P379/385) was associated with sequence variation in *Tpi*‐intron4 (Figure 4; Figure S2), which could further be developed as a marker to subdivide C‐strain samples. It is noteworthy that a particular (Africa‐specific) haplotype of the *Tpi* gene originally identified in Africa was tentatively designated as R‐strain based on the E4^183^ site (equal to P370 in Figure 4 in this study) in previous studies (Nagoshi, 2012). Our genome‐wide SNP analysis revealed that this haplotype contained more C‐strain SNPs than R‐strain SNPs.

The sample used for the genome sequencing in this study represents a combination of the particular *Tpi* haplotype and C‐strain *COI*. We also found combinations of the R‐strain *COI* and particular *Tpi* (sample G‐XW13), as well as heterozygous forms of the particular *Tpi* and *Tpi‐*C with the R‐strain *COI* in two samples (G‐GXW11, G‐EP6). These combinations of different genotypes show that the genetic boundaries between two established (American) R‐ and C‐strains are obscure. The insertion of two mitochondrial fragments into the nuclear genome might be caused by random hybridization between different genotypes, which would suggest that fall armyworm invading China might be descendants of an interstrain hybrid population. This is the first report of DNA fragments transferred from mitochondria into the nuclear genome in a *Spodoptera* lineage, and two such fragments could be used to develop markers to identify specific populations and to follow further evolutionary events of fall armyworm.

The rapid evolution of insecticide resistance and the increasing levels of resistance observed in fall armyworm populations needs attention. In this study, reported mutations related to insecticide resistance were detected in the AChE gene. Although some mutation sites were detected as heterozygous in most samples, the frequency of resistant mutation sites will increase greatly under the selection pressure caused by application of related pesticides in the field. The bioassay results showed that armyworms invading China have evolved high levels of resistance to organophosphate pesticides, which was consistent with the results of molecular scanning of resistance‐related genes, yet the resistance to pyrethroid pesticides cannot be explained by any reported mechanism. However, the fall armyworms invading China are currently sensitive to GM maize expressing Cry1Ab in field experiments, and are also sensitive to other Bt toxins in the laboratory, according to previous studies (Li et al., 2019). At present, GM maize shows better application prospects in controlling fall armyworm in China, as larval density and damage rate of GM maize were significantly less than that of non‐GM plants, although this crop is currently not registered for use in China.

This study provides a high‐quality reference genome that demonstrates a genomic feature different from the established (American) C‐ or R‐strain genotypes, as well as more comprehensive gene annotation. We also present resequencing data for 103 fall armyworm individuals invading China. The samples cover different regions and times during 2019, providing basic materials for analysing global population genetic and identifying patterns of invasiveness. Baseline resistance data for Chinese fall armyworm populations are shown to 14 common pesticides, providing guidance for the control and resistance monitoring of fall armyworm. Small‐scale field experiments in this study suggest that fall armyworm in China are currently susceptible to GM maize, and these results could provide an important application reference for commercial planting of Bt maize in China. There are other important issues that remain for further exploitation using this whole genome approach, such as identifying the genes involved in polyphagy, migratory capability and olfaction, which could provide valuable tools for the future management of fall armyworms.

## AUTHOR CONTRIBUTIONS

Y.X., K.W., W.Q. and W.F. conceived the project, designed content and managed the project; L.Z. and G.W. coordinated the project; B.L., Z.L. and X. Liu. performed read mapping, SNP calling and population analysis; W.Z. and C.L. performed genome assembly and annotation; L.Z. and B.L. performed Hi‐C assembly; D.Z. performed the laboratory bioassay; S.Z. performed the field experiment; P.X. performed transcriptome analysis; K.N. and E.A. provided the raw data of American R‐ and C‐strains; B. Liu., X.L., M.J., C.W. and X.Y. performed the DNA extraction, PCR and sequence variation analysis; W.Q. constructed DNA libraries, performed sequencing; Y.J. and J.L. collected and provided samples from China; L.Z. wrote the manuscript; K.W., A.W., C.M.J., J.A.S., G.C., D.L.K. and S.C. revised the manuscript. All authors commented on the manuscript.

## Supporting information

Supplementary Material

## Data Availability

This Whole Genome Shotgun project of *Spodoptera frugiperda* has been deposited at DDBJ/ENA/GenBank under accession no. WUTJ00000000 with BioProject ID PRJNA591441. The version described in this paper is version WUTJ01000000. Raw sequencing reads of PacBio, RNA‐seq, Hi‐C and resequencing in this paper can be accessed at ftp://ftp.agis.org.cn/Spodoptera_Frugiperda/.
