# Supplementary material for: Genetic structure and insecticide resistance characteristics of fall armyworm populations invading China
Source: Mol Ecol Resour. 2020 Jul 20;20(6):1682–96. doi: 10.1111/1755-0998.13219 (PMC7689805; doi:10.1111/1755-0998.13219)
Supplement: Supplementary file 1 — Supplementary Material [file MEN-20-1682-s001.pdf]

MOLECULAR ECOLOGY RESOURCES

Supplemental Information for:

Genetic structure and insecticide resistance characteristics of fall armyworm populations invading China

Lei Zhang[1#], Bo Liu[1#], Weigang Zheng[1#], Conghui Liu[1#], Dandan Zhang[2#], Shengyuan Zhao[2#], Zaiyuan Li[1], Pengjun Xu[3,4], Kenneth Wilson[4,1], Amy Withers[4], Christopher M. Jones[5], Judith A. Smith[6], Gilson Chipabika[7], Donald L. Kachigamba[8], Kiwoong Nam[9], Emmanuelle d'Alençon[9], Bei Liu[1], Xinyue Liang[1], Minghui Jin[1], Chao Wu[1], Swapan Chakrabarty[1], Xianming Yang[2], Yuying Jiang[10], Jie Liu[10], Xiaolin Liu[11], Weipeng Quan[12], Guirong Wang[1], Wei Fan[1*], Wanqiang Qian[1*], Kongming Wu[2*], Yutao Xiao[1*]

[#]These authors contributed equally to this work.
[*]Correspondence: Yutao Xiao (xiaoyutao@caas.cn), Kongming Wu (wukongming@caas.cn), Wanqiang Qian (qianwanqiang@caas.cn), Wei Fan (fanwei@caas.cn).

Table of Contents:

Supplementary Table 1. Information of 107 re-sequenced fall armyworm samples in this study.	Page 1-3
Supplementary Table 2. Information of 173 fall armyworm samples used for population genetic analysis in this study.	Page 4-11
Supplementary Table 3. Primer sequence information used in this study.	Page 12
Supplementary Table 4. General information of 14 kinds of insecticides used in topical bioassays.	Page 13
Supplementary Table 5. Comparison between two versions of published fall armyworm genomes and the genome of this study.	Page 13
Supplementary Table 6. Annotation and distribution of repetitive elements in fall armyworm genome.	Page 14
Supplementary Table 7. Summary of genome assemblies used in this study.	Page 15
Supplementary Table 8. Genes related to pesticide resistance and Bt resistance in fall armyworm.	Page 16
Supplementary Table 9. Distribution of mitochondrial insertion in 107 re-sequenced fall armyworm samples and American corn-strain (ASW) and rice-strain (AXE).	Page 17-21
Supplementary Figure 1. Map of fall armyworm collection sites in China.	Page 22
Supplementary Figure 2. Comparison of different Tpi genotypes in Chinese samples.	Page 23

Supplementary Table 1. Information of 107 re-sequenced fall armyworm samples in this study.

Sample No.	Collection sites	Collection time	Stages	Host or source	Numbers
AFR4, AFR5	Lusaka, Zambia	2017-03-01	Larva	Corn	2
AFR14, AFR15	Bvumbwe, Malawi	2019-01-01	Larva	Corn	2
YKM1, YKM2	Kunming City, Yunnan Province	2019-05-27	Larva	Corn	2
GHZ1, GHZ2	Hezhou City, Guangxi Province	2019-05-13	Larva	Corn	2
GBL1	Yulin City, Guangxi Province	2019-05-30	Larva	Corn	1
GLZ1, GLZ3	Liuzhou City, Guangxi Province	2019-06-17	Larva	Sugarcane	2
HY1, HY2	Heyuan City, Guangdong Province	2019-05-08	Larva	Corn	2
FS1, FS2	Foshan City, Guangdong Province	2019-05-10	Larva	Corn	2
XW1, XW2	Zhanjiang City, Guangdong Province	2019-05-10	Larva	Corn	2
DG1, DG2	Dongguan City, Guangdong Province	2019-05-13	Larva	Corn	2
HZ1, HZ2	Huizhou City, Guangdong Province	2019-05-13	Larva	Corn	2
ZH1, ZH2	Zhuhai City, Guangdong Province	2019-05-13	Larva	Corn	2
WC1, WC2	Zhanjiang City, Guangdong Province	2019-05-14	Larva	Corn	2
YF1, YF2	Yunfu City, Guangdong Province	2019-05-19	Larva	Corn	2
SG1, SG2	Shaoguan City, Guangdong Province	2019-05-27	Larva	Corn	2
GHZ2-1	Huizhou City, Guangdong Province	2019-06-09	Larva	Corn	1
GWY1, GWY2	Shaoguan City, Guangdong Province	2019-06-09	Larva	Corn	2
GNS1, GNS2	Guangzhou City, Guangdong Province	2019-06-13	Larva	Corn	2
GQY1, GQY2	Qingyuan City, Guangdong Province	2019-06-13	Larva	Corn	2
GLD1	Luoding City, Guangdong Province	2019-06-16	Larva	Corn	1
GFS1, GFS2	Foshan City, Guangdong Province	2019-06-17	Larva	Corn	2
GJX11, GJX12, GJX13	Jieyang City, Guangdong Province	2019-06-20	Larva	Corn	3

GJX15, GJX16	Jieyang City, Guangdong Province	2019-06-28	Larva	Corn	2
YZ1, YZ2	Yongzhou City, Hunan Province	2019-05-08	Larva	Corn	2
CZ1, CZ2	Chenzhou City, Hunan Province	2019-05-10	Larva	Corn	2
XP1, XP2	Huaihua City, Hunan Province	2019-05-15	Larva	Corn	2
HSY1-BF	Shaoyang City, Hunan Province	2019-05-31	Larva	Corn	1
HN1	Haiko City, Hainan Province	2019-05-08	Larva	Corn	1
HSY1, HSY2	Sanya City, Hainan Province	2019-06-03	Larva	Corn	2
ZLS2	Lishui City, Zhejiang Province	2019-05-27	Larva	Corn	1
ZLQ1	Leqing City, Zhejiang Province	2019-06-03	Larva	Corn	1
SHZ1	Hanzhong City, Shanxi Province	2019-06-06	Larva	Corn	1
SXX1	Hanzhong City, Shanxi Province	2019-06-09	Larva	Corn	1
JJA1, JJA2	Jian City, Jiangxi Province	2019-06-09	Larva	Corn	2
JPX1, JPX2	Pingxiang City, Jiangxi Province	2019-06-09	Larva	Corn	2
JHK1	Huko City, Jiangxi Province	2019-06-12	Larva	Corn	1
JJJ1, JJJ2, JJJ3, JJJ4	Jiujiang City, Jiangxi Province	2019-06-13	Larva	Corn	4
JNC1, JNC2	Nanchang City, Jiangxi Province	2019-06-13	Larva	Corn	2
JNC3, JNC4	Nanchang City, Jiangxi Province	2019-06-13	Larva	Corn	2
JXY1, JXY2	Xinyu City, Jiangxi Province	2019-06-16	Larva	Corn	2
JJJ11, JJJ12	Jiujiang City, Jiangxi Province	2019-06-17	Larva	Corn	2
JNC12	Nanchang City, Jiangxi Province	2019-06-17	Larva	Corn	1
SFX1	Fengxian District, Shanghai	2019-06-09	Larva	Corn	1
HJZ2	Jingzhou City, Hubei Province	2019-05-11	Larva	Corn	1
HXG1, HXG2	Xiaogan City, Hubei Province	2019-06-10	Larva	Corn	2
HTS1, HTS2	Xianning City, Hubei Province	2019-06-13	Larva	Corn	2
HYC1, HYC2	Yichang City, Hubei Province	2019-06-13	Larva	Corn	2

HXZ1, HXZ2	Wuhan District, Hubei Province	2019-06-26	Larva	Corn	2
CWX1, CWX2	Wuxi Country, Chongqing	2019-06-09	Larva	Corn	2
CXS1, CXS2	Xiushan Country, Chongqing	2019-06-09	Larva	Corn	2
CQJ2	Qianjiang District, Chongqing	2019-06-21	Larva	Corn	1
XLZ1	Linzhi City, Xizang Province	2019-06-16	Larva	Corn	1
AWJ1, AWJ2	Anqing City, Anhui Province	2019-06-19	Larva	Corn	2
AWH1, AWH2	Wuhu City, Anhui Province	2019-06-20	Larva	Corn	2
AAQ1, AAQ2	Anqing City, Anhui Province	2019-06-24	Larva	Corn	2
ACZ1, ACZ2, ACZ3, ACZ4	Chuzhou City, Anhui Province	2019-06-24	Larva	Corn	4
JHM1	Nantong City, Jiangsu Province	2019-06-24	Larva	Corn	1
GGY1, GGY2	Guiyang City, Guizhou Province	2019-06-26	Larva	Corn	2
SLS1, SLS2	Liangshan State, Sichuan Province	2019-06-26	Larva	Corn	2
				Total	107

Supplementary Table 2. Information of 173 fall armyworm samples used for population genetic analysis in this study.

Serial No.	Collection site	Sample No.	Collection time	Stages	Host or source	*COI*	Tpi	Insertion
1	Longnan City, Gansu Province	G-GLN5	2019-07-15	Larva	Corn	Rice strain	Corn strain	×
2	Longnan City, Gansu Province	G-GLN6	2019-07-15	Larva	Corn	Rice strain	Corn strain	×
3	Longnan City, Gansu Province	G-GLN7	2019-07-15	Larva	Corn	Rice strain	Corn strain	×
4	Longnan City, Gansu Province	G-GLN8	2019-07-15	Larva	Corn	Rice strain	Corn strain	√
5	Hechi City, Guangxi Province	G-GHC15	2019-07-01	Adult	Corn	Rice strain	Corn strain	×
6	Hechi City, Guangxi Province	G-GHC16	2019-07-01	Adult	Corn	Corn strain	Corn strain	√
7	Xuancheng City, Anhui Province	G-AJD1	2019-07-01	Larva	Corn	Rice strain	Corn strain	×
8	Xuancheng City, Anhui Province	G-AJD2	2019-07-01	Larva	Corn	Rice strain	Corn strain	×
9	Xuancheng City, Anhui Province	G-AXC6	2019-07-01	Larva	Corn	Rice strain	Corn strain	×
10	Xuancheng City, Anhui Province	G-AXC7	2019-07-01	Larva	Corn	Rice strain	Corn strain	×
11	Liangshan State, Sichuan Province	G-SLS1	2019-07-01	Larva	Corn	Rice strain	Corn strain	×
12	Liangshan State, Sichuan Province	G-SLS2	2019-07-01	Larva	Corn	Rice strain	Corn strain	×
13	Huangshan City, Anhui Province	G-AHS1	2019-06-28	Larva	Corn	Rice strain	Corn strain	√
14	Huangshan City, Anhui Province	G-AHS2	2019-06-28	Larva	Corn	Rice strain	Corn strain	×
15	Chaozhou City, Guangdong Province	G-GCZ1	2019-06-28	Larva	Corn	Rice strain	Corn strain	×
16	Chaozhou City, Guangdong Province	G-GCZ2	2019-06-28	Larva	Corn	Rice strain	Corn strain	√
17	Tongcheng City, Anhui Province	G-ATC3	2019-06-28	Larva	Corn	Rice strain	Corn strain	×
18	Tongcheng City, Anhui Province	G-ATC4	2019-06-28	Larva	Corn	Rice strain	Corn strain	√
19	Jieyang City, Guangdong Province	G-GJX10	2019-06-28	Larva	Corn	Rice strain	Corn strain	×
20	Jieyang City, Guangdong Province	G-GJX11	2019-06-28	Larva	Corn	Rice strain	Corn strain	√

21	Hechi City, Guangxi Province	G-GDH1	2019-06-24	Adult	Corn	Rice strain	Corn strain	×
22	Hechi City, Guangxi Province	G-GDH2	2019-06-24	Adult	Corn	Rice strain	Corn strain	×
23	Anqing City, Anhui Province	G-AAQ1	2019-06-24	Larva	Corn	Rice strain	Corn strain	×
24	Anqing City, Anhui Province	G-AAQ2	2019-06-24	Larva	Corn	Rice strain	Corn strain	×
25	Xinyu City, Jiangxi Province	G-JXY3	2019-06-17	Larva	Corn	Rice strain	Corn strain	×
26	Xinyu City, Jiangxi Province	G-JXY4	2019-06-17	Larva	Corn	Rice strain	Corn strain	×
27	Linzhi City, Xizang Province	G-XLZ1	2019-06-17	Larva	Corn	Rice strain	Corn strain	√
28	Linzhi City, Xizang Province	G-XLZ2	2019-06-17	Larva	Corn	Rice strain	Corn strain	√
29	Yunfu City, Guangdong Province	G-GLD1	2019-06-17	Adult	Corn	Rice strain	Corn strain	×
30	Yunfu City, Guangdong Province	G-GLD2	2019-06-17	Adult	Corn	Rice strain	Corn strain	×
31	Xiaogan City, Hubei Province	G-HXG1	2019-06-17	Larva	Corn	Rice strain	Corn strain	√
32	Xiaogan City, Hubei Province	G-HXG2	2019-06-17	Larva	Corn	Rice strain	Corn strain	√
33	Jiujiang City, Jiangxi Province	G-JHK1	2019-06-17	Larva	Corn	Rice strain	Corn strain	×
34	Jiujiang City, Jiangxi Province	G-JHK2	2019-06-17	Larva	Corn	Rice strain	Corn strain	×
35	Nanchang City, Jiangxi Province	G-JNC11	2019-06-17	Larva	Corn	Rice strain	Corn strain	×
36	Nanchang City, Jiangxi Province	G-JNC12	2019-06-17	Larva	Corn	Rice strain	Corn strain	×
37	Foshan City, Guangdong Province	G-GFS2	2019-06-17	Adult	Corn	Corn strain	Corn strain	×
38	Foshan City, Guangdong Province	G-GFS3	2019-06-17	Egg mass	Corn	Corn strain	Corn strain	×
39	Nanchang City, Jiangxi Province	G-JNC1	2019-06-13	Larva	Corn	Rice strain	Corn strain	×
40	Nanchang City, Jiangxi Province	G-JNC2	2019-06-13	Larva	Corn	Rice strain	Corn strain	×
41	Nanchang City, Jiangxi Province	G-JNC3	2019-06-13	Larva	Corn	Corn strain	Corn strain	×
42	Nanchang City, Jiangxi Province	G-JNC4	2019-06-13	Pupa	Corn	Rice strain	Corn strain	×
43	Qingyuan City, Guangdong Province	G-EMS1	2019-06-13	Larva	Corn	Rice strain	Corn strain	×
44	Qingyuan City, Guangdong Province	G-EMS2	2019-06-13	Larva	Corn	Rice strain	Corn strain	×
45	Yichang City, Hubei Province	G-HYC1	2019-06-13	Larva	Corn	Rice strain	Corn strain	×

46	Yichang City, Hubei Province	G-HYC2	2019-06-13	Larva	Corn	Rice strain	Corn strain	×
47	Xinyu City, Jiangxi Province	G-JXY1	2019-06-13	Larva	Corn	Rice strain	Corn strain	√
48	Xinyu City, Jiangxi Province	G-JXY2	2019-06-13	Larva	Corn	Rice strain	Corn strain	×
49	Xianning City, Hubei Province	G-HTS1	2019-06-13	Larva	Corn	Rice strain	Corn strain	×
50	Xianning City, Hubei Province	G-HTS2	2019-06-13	Larva	Corn	Rice strain	Corn strain	×
51	Jiujiang City, Jiangxi Province	G-JJJ1	2019-06-13	Larva	Corn	Rice strain	Corn strain	×
52	Jiujiang City, Jiangxi Province	G-JJJ2	2019-06-13	Larva	Corn	Rice strain	Corn strain	×
53	Pingxiang City, Jiangxi Province	G-PX1	2019-06-10	Larva	Corn	Rice strain	Corn strain	√
54	Pingxiang City, Jiangxi Province	G-PX2	2019-06-10	Larva	Corn	Rice strain	Corn strain	√
55	Wuxi Country, Chongqing City	G-WX1	2019-06-10	Larva	Corn	Rice strain	Corn strain	√
56	Wuxi Country, Chongqing City	G-WX2	2019-06-10	Larva	Corn	Rice strain	Corn strain	√
57	Jian City, Jiangxi Province	G-JA1	2019-06-10	Larva	Corn	Rice strain	Corn strain	√
58	Jian City, Jiangxi Province	G-JA2	2019-06-10	Larva	Corn	Rice strain	Corn strain	×
59	Yunfu City, Guangdong Province	G-YF13	2019-05-31	Larva	Corn	Rice strain	Corn strain	×
60	Yunfu City, Guangdong Province	G-YF14	2019-05-31	Larva	Corn	Rice strain	Corn strain	×
61	Zhaoqing City, Guangdong Province	G-ZQ5	2019-05-31	Larva	Corn	Rice strain	Corn strain	×
62	Zhaoqing City, Guangdong Province	G-ZQ6	2019-05-31	Larva	Corn	Rice strain	Corn strain	√
63	Shaoyang City, Hunan Province	G-HSY8	2019-05-31	Larva	Corn	Rice strain	Corn strain	×
64	Yunfu City, Guangdong Province	G-GYF1	2019-05-31	Adult	Corn	Rice strain	Corn strain	√
65	Zhanjiang City, Guangdong Province	G-XS1	2019-05-24	Larva	Corn	Rice strain	Corn strain	×
66	Zhanjiang City, Guangdong Province	G-XS2	2019-05-24	Larva	Corn	Rice strain	Corn strain	×
67	Yiyang City, Hunan Province	G-YY1	2019-05-24	Larva	Corn	Rice strain	Corn strain	×
68	Yiyang City, Hunan Province	G-YY2	2019-05-24	Larva	Corn	Rice strain	Corn strain	×
69	Yongzhou City, Hunan Province	G-EM1	2019-05-24	Larva	Corn	Rice strain	Corn strain	×
70	Yongzhou City, Hunan Province	G-EM2	2019-05-24	Adult	Corn	Rice strain	Corn strain	×

71	Yunfu City, Guangdong Province	G-YF10	2019-05-24	Larva	Corn	Rice strain	Corn strain	√
72	Yunfu City, Guangdong Province	G-YF11	2019-05-24	Larva	Corn	Rice strain	Corn strain	×
73	Meizhou City, Guangdong Province	G-PY1	2019-05-24	Larva	Corn	Rice strain	Corn strain	√
74	Meizhou City, Guangdong Province	G-PY2	2019-05-24	Larva	Corn	Rice strain	Corn strain	×
75	Meizhou City, Guangdong Province	G-DP1	2019-05-23	Larva	Corn	Rice strain	Corn strain	×
76	Meizhou City, Guangdong Province	G-DP2	2019-05-23	Larva	Corn	Rice strain	Corn strain	×
77	Meizhou City, Guangdong Province	G-WH1	2019-05-23	Larva	Corn	Rice strain	Corn strain	×
78	Meizhou City, Guangdong Province	G-WH2	2019-05-23	Larva	Corn	Rice strain	Corn strain	×
79	Loudi City, Hunan Province	G-LG1	2019-05-21	Larva	Corn	Rice strain	Corn strain	×
80	Loudi City, Hunan Province	G-LG2	2019-05-21	Larva	Corn	Rice strain	Corn strain	×
81	Hengyang City, Hunan Province	G-HY1	2019-05-21	Larva	Corn	Rice strain	Corn strain	×
82	Hengyang City, Hunan Province	G-HY2	2019-05-21	Larva	Corn	Rice strain	Corn strain	×
83	Huaihua City, Hunan Province	G-HH5	2019-05-21	Larva	Corn	Rice strain	Corn strain	√
84	Huaihua City, Hunan Province	G-HH6	2019-05-21	Larva	Corn	Corn strain	Corn strain	×
85	Yunfu City, Guangdong Province	G-YF1	2019-05-21	Larva	Corn	Rice strain	Corn strain	×
86	Yunfu City, Guangdong Province	G-YF2	2019-05-21	Larva	Corn	Rice strain	Corn strain	×
87	Haiko City, Hainan Province	G-HN1	2019-05-20	Larva	Corn	Rice strain	Corn strain	×
88	Haiko City, Hainan Province	G-HN2	2019-05-20	Larva	Corn	Rice strain	Corn strain	×
89	Shaoyang City, Hunan Province	G-HSY20	2019-05-17	Larva	Corn	Rice strain	Corn strain	×
90	Shaoyang City, Hunan Province	G-HSY21	2019-05-17	Larva	Corn	Rice strain	Corn strain	√
91	Shaoyang City, Hunan Province	G-HSY22	2019-05-17	Larva	Corn	Rice strain	Corn strain	×
92	Shaoyang City, Hunan Province	G-HSY23	2019-05-17	Larva	Corn	Rice strain	Corn strain	×
93	Huaihua City, Hunan Province	G-HH3	2019-05-17	Larva	Corn	Rice strain	Corn strain	×
94	Huaihua City, Hunan Province	G-HH4	2019-05-17	Larva	Corn	Rice strain	Corn strain	×
95	Changde City, Hunan Province	G-CD1	2019-05-17	Larva	Corn	Rice strain	Corn strain	√

96	Changde City, Hunan Province	G-CD2	2019-05-17	Larva	Corn	Rice strain	Corn strain	√
97	Heyuan City, Guangdong Province	G-DY1	2019-05-17	Larva	Corn	Rice strain	Corn strain	×
98	Heyuan City, Guangdong Province	G-DY2	2019-05-17	Larva	Corn	Rice strain	Corn strain	×
99	Huaihua City, Hunan Province	G-HH1	2019-05-17	Larva	Corn	Rice strain	Corn strain	×
100	Huaihua City, Hunan Province	G-HH2	2019-05-17	Larva	Corn	Rice strain	Corn strain	×
101	Miluo City, Hunan Province	G-JL1	2019-05-17	Larva	Corn	Rice strain	Corn strain	×
102	Miluo City, Hunan Province	G-JL2	2019-05-17	Larva	Corn	Rice strain	Corn strain	√
103	Maoming City, Guangdong Province	G-MM6	2019-05-17	Larva	Corn	Rice strain	Corn strain	×
104	Maoming City, Guangdong Province	G-MM7	2019-05-17	Larva	Corn	Rice strain	Corn strain	×
105	Yongzhou City, Hunan Province	G-YZ1	2019-05-17	Larva	Corn	Rice strain	Corn strain	×
106	Yongzhou City, Hunan Province	G-YZ2	2019-05-17	Larva	Corn	Rice strain	Corn strain	√
107	Dongguan City, Guangdong Province	G-DW6	2019-05-17	Larva	Corn	Rice strain	Corn strain	×
108	Dongguan City, Guangdong Province	G-DW7	2019-05-17	Larva	Corn	Rice strain	Corn strain	×
109	Huizhou City, Guangdong Province	G-GHZ15	2019-05-17	Adult	Corn	Rice strain	Corn strain	√
110	Huizhou City, Guangdong Province	G-GHZ16	2019-05-17	Adult	Corn	Rice strain	Corn strain	×
111	Chenzhou City, Hunan Province	G-CZ1	2019-05-14	Larva	Corn	Rice strain	Corn strain	×
112	Chenzhou City, Hunan Province	G-CZ2	2019-05-14	Larva	Corn	Rice strain	Corn strain	√
113	Zhanjiang City, Guangdong Province	G-WC1	2019-05-14	Larva	Corn	Rice strain	Corn strain	×
114	Zhanjiang City, Guangdong Province	G-WC2	2019-05-14	Larva	Corn	Rice strain	Corn strain	×
115	Changde City, Hunan Province	G-HCD1	2019-05-14	Larva	Corn	Rice strain	Corn strain	×
116	Shaoyang City, Hunan Province	G-SY5	2019-05-14	Larva	Corn	Rice strain	Corn strain	×
117	Yongzhou City, Hunan Province	G-HYZ1	2019-05-14	Larva	Corn	Rice strain	Corn strain	√
118	Yongzhou City, Hunan Province	G-HYZ2	2019-05-14	Larva	Corn	Rice strain	Corn strain	×

119	Shantou City, Guangdong Province	G-GST1	2019-07-06	Larva	Corn	Corn strain	Corn strain	√
120	Shantou City, Guangdong Province	G-GST2	2019-07-06	Larva	Corn	Corn strain	Corn strain	√
121	Anqing City, Anhui Province	G-AWJ2	2019-06-26	Larva	Corn	Corn strain	Corn strain	√
122	Shaoguan City, Guangdong Province	G-WY1	2019-06-11	Larva	Corn	Corn strain	Corn strain	×
123	Jieyang City, Guangdong Province	G-JX2	2019-05-22	Larva	Corn	Corn strain	Corn strain	×
124	Zhanjiang City, Guangdong Province	G-ZJ3	2019-05-08	Larva	Corn	Corn strain	Corn strain	×
125	Zhanjiang City, Guangdong Province	G-ZJ4	2019-05-08	Larva	Corn	Corn strain	Corn strain	√
126	Yongzhou City, Hunan Province	G-YZ4	2019-05-06	Larva	Corn	Corn strain	Corn strain	×
127	Yongzhou City, Hunan Province	G-YZ3	2019-05-06	Larva	Corn	Corn strain	Corn strain	×
128	Yongzhou City, Hunan Province	G-JM2	2019-05-06	Larva	Corn	Corn strain	Corn strain	√
129	Yongzhou City, Hunan Province	G-JM3	2019-05-06	Larva	Corn	Corn strain	Corn strain	√
130	Qinzhou City, Guangxi Province	G-PB7	2019-04-29	Larva	Corn	Corn strain	Corn strain	√
131	Qinzhou City, Guangxi Province	G-GLS7	2019-08-19	Larva	Rice	Rice strain	Corn strain	×
132	Qinzhou City, Guangxi Province	G-GLS8	2019-08-19	Larva	Rice	Rice strain	Corn strain	×
133	Liuzhou City, Guangxi Province	G-GLZ1	2019-06-17	Larva	Sugar cane	Rice strain	Corn strain	×
134	Lingao Country, Hainan Province	G-GLG3	2019-07-17	Larva	Zedoary	Rice strain	Corn strain	×
135	Zhanjiang City, Guangdong Province	**G-GXW11**	2019-07-29	Larva	Corn	Rice strain	**Africa-specific/Corn**	√
136	Zhanjiang City, Guangdong Province	**G-GXW13**	2019-08-13	Larva	Corn	Rice strain	**Africa-specific**	×
137	Jiangmen City, Guangdong Province	**G-EP6**	2019-04-29	Larva	Corn	Rice strain	**Africa-specific/Corn**	√
138	Tengzhou City, Shandong Province	G-STZ3	2019-07-30	Larva	Corn	Rice strain	Corn strain	√
139	Tengzhou City, Shandong Province	G-STZ4	2019-07-30	Larva	Corn	Rice strain	Corn strain	×
140	Ruili City, Yunnan Province	G-RL1	2019-07-30	Larva	Corn	Rice strain	Corn strain	×
141	Ruili City, Yunnan Province	G-RL2	2019-07-30	Larva	Corn	Rice strain	Corn strain	×
142	Yongcheng City, Henan Province	G-HYC3	2019-08-05	Larva	Corn	Rice strain	Corn strain	×
143	Yongcheng City, Henan Province	G-HYC4	2019-08-05	Larva	Corn	Rice strain	Corn strain	√

144	Luoyang City, Henan Province	G-HLY2	2019-07-29	Larva	Corn	Rice strain	Corn strain	√
145	Nanyang City, Henan Province	G-HNY2	2019-07-29	Larva	Corn	Rice strain	Corn strain	×
146	Xian City, Shanxi Province	G-SXA1	2019-07-29	Larva	Corn	Rice strain	Corn strain	×
147	Xian City, Shanxi Province	G-SXA2	2019-07-29	Larva	Corn	Rice strain	Corn strain	×
148	Guiyang City, Guizhou Province	G-GGY1	2019-06-27	Larva	Corn	Rice strain	Corn strain	×
149	Guiyang City, Guizhou Province	G-GGY2	2019-06-27	Larva	Corn	Rice strain	Corn strain	×
150	Nantong City, Jiangsu Province	G-JHM1	2019-06-24	Larva	Corn	Rice strain	Corn strain	×
151	Nantong City, Jiangsu Province	G-JHM2	2019-06-24	Larva	Corn	Rice strain	Corn strain	×
152	Fengxian District, Shanghai City	G-FX1	2019-06-09	Larva	Corn	Rice strain	Corn strain	×
153	Wenzhou City, Zhejiang Province	G-LQ1	2019-06-03	Larva	Corn	Rice strain	Corn strain	×
154	Ningbo City, Zhejiang Province	G-ZLB2	2019-06-03	Larva	Corn	Rice strain	Corn strain	×
155	Kunming City, Yunnan Province	G-KM2	2019-05-27	Larva	Corn	Rice strain	Corn strain	√
156	Ningde City, Fujian Province	G-LD1	2019-05-14	Larva	Corn	Rice strain	Corn strain	×
157	Zhanjiang City, Guangdong Province	G-GLZ6	2019-08-27	Larva	Corn	Rice strain	Corn strain	√
158	Zhanjiang City, Guangdong Province	G-GLZ7	2019-08-27	Larva	Corn	Corn strain	Corn strain	×
159	Handan City, Hebei Province	G-HHD5	2019-08-27	Larva	Corn	Rice strain	Corn strain	×
160	Handan City, Hebei Province	G-HHD6	2019-08-27	Larva	Corn	Rice strain	Corn strain	×
161	Yulin City, Guangxi Province	G-GYL1	2019-08-27	Larva	Rice	Rice strain	Corn strain	×
162	Yulin City, Guangxi Province	G-GYL2	2019-08-27	Larva	Rice	Rice strain	Corn strain	×
163	Handan City, Hebei Province	G-HHD7	2019-08-27	Larva	Corn	Rice strain	Corn strain	×
164	Handan City, Hebei Province	G-HHD8	2019-08-27	Larva	Corn	Rice strain	Corn strain	√
165	Baiyin City, Gansu Province	G-GBY1	2019-08-27	Larva	Corn	Rice strain	Corn strain	√
166	Baiyin City, Gansu Province	G-GBY2	2019-08-27	Larva	Corn	Rice strain	Corn strain	×
167	Xingtai City, Hebei Province	G-HXT1	2019-08-27	Larva	Corn	Rice strain	Corn strain	×
168	Xingtai City, Hebei Province	G-HXT3	2019-08-27	Larva	Corn	Rice strain	Corn strain	×

169	Xingtai City, Hebei Province	G-HXT4	2019-08-27	Larva	Corn	Rice strain	Corn strain	×
170	Liaocheng City, Shandong Province	G-SLC1	2019-08-27	Larva	Corn	Rice strain	Corn strain	×
171	Liaocheng City, Shandong Province	G-SLC2	2019-08-27	Larva	Corn	Rice strain	Corn strain	×
172	Handan City, Hebei Province	G-HHD9	2019-08-27	Larva	Corn	Rice strain	Corn strain	×
173	Handan City, Hebei Province	G-HHD1	2019-08-27	Larva	Corn	Corn strain	Corn strain	×
						19 (10.98%)	3 (0.02%)	45 (26.01%)

Supplementary Table 3. Primer sequence information used in this study.

Primer	Sequence (5' -3')	Target	Reference
CO1-F	TTCGAGCTGAATTAGGGACTC	*COI* gene	Nagoshi, et al. 2017
CO1-R	GATGTAAAATATGCTCGTGT		
Tpi-F	GGTGAAATCTCCCCTGCTATG	Tpi gene	Nagoshi, et al. 2017
RR1	GATTACTGAAACCAATTCTGATT		This study
GAP1-F2	CGGTACATAGACTGGTCG	Gap1	This study
GAP1-R2	CAGTTGCGGTTAAACAAA		
GAP2-F123	TTTCAACAGAAGCCTCAT	Gap2	This study
GAP2-R3	AACACCTAACCCTACTCA		
GAP3-F3	TGAATGGGTATCCAGAGC	Gap3	This study
GAP3-R13	CCAATGAATGGTGGTAAT		
GAP4-F3	ATTGCTCATGGTGGTAGT	Gap4	This study
GAP4-R3	GAGCCATCCTTCAATACA		
LzSF2_F	TATTTCACCCGACACTCA	*ABCC2*	This study
LzSF2_R	TCCATCCACCTGGTTAGT		
AceF2Lep	AATCCCAACACAGACATGC	AChE	Carvalho, et al. 2013
AceSf R1	ATAACTTACCCGGTCCTCGC		

Supplementary Table 4. General information of 14 kinds of insecticides used in topical bioassays.

Variety	Insecticides	Purity (%)
Pyrethroids	Beta-cypermethrin	94.3
	Fenvalerate	97.5
	Deltamethrin	86
	Lambda-cyhalothrin	99.1
Organophosphates	Malathion	95
	Chlorpyrifos	97
	Phoxim	98.6
Carbamate	Indoxacarb	70
Amide	Chlorantraniliprole	97
	Cyantraniliprole	95
Microbial insecticides	Spinosad	53
	Emamectin benzoate	72
Others	E-MBI	12
	Chlorfenapyr	98

Supplementary Table 5. Comparison between two versions of published fall armyworm genomes and the genome of this study.

Comparasion	Genome version	multiple alignment	Unique alignment	Reference
VS Corn-strain	This study	99.71%	99.37%	This study
	Corn-strain	98.08%	91.01%	Gouin et al., 2017
VS Rice-strain	This study	98.94%	98.76%	This study
	Rice-strain	98.70%	93.92%	Gouin et al., 2017
VS Male moth	This study	99.05%	98.98%	This study
	Male moth	99.33%	89.75%	Liu et al., 2019
VS Female moth	This study	99.04%	98.99%	This study
	Female moth	98.87%	88.70%	Liu et al., 2019

Supplementary Table 6. Annotation and distribution of repetitive elements in fall armyworm genome.

Family	Number of Repeat	Length(bp)	percentage
DNA_known	50953	10725623	2.73%
DNA_unknown	20	1102	0.00%
DNA_virus	1	51	1.30E-07
LINE_known	165258	34044870	8.66%
LINE_unknown	1	71	1.81E-07
LTR_known	34468	5418871	1.38%
LTR_unknown	1	48	1.22E-07
Low_complexity	21	4216	0.00%
RC	23160	4861285	1.24%
RNA	108	12885	0.00%
Retroposon_known	1	62	1.58E-07
Retroposon_unknown	1	39	9.92E-08
SINE_known	24273	3511475	0.89%
SINE_unknown	3820	485583	0.12%
Satellite	509	84450	0.02%
Simple_repeat_known	2375	392372	0.10%
Simple_repeat_Unknown	275517	47272655	12.02%
rRNA	116	52287	0.01%
snRNA	95	12227	0.00%
tRNA	280	21260	0.01%
Other	6	1068	0.00%
Total	580984	106902500	27.18%

Supplementary Table 7. Summary of genome assemblies used in this study.

Species name	Genome size (Mb)	N50 of scaffold (kb)	GC content (%)	Number of gene models	BUSCO complete gene (%)	Source
Spodoptera frugiperda	390	12966.7	36.4	22,260	98.4	This study
Spodoptera litura	439	13592.0	36.6	16,169	99.4	PRJNA344815<br>GenBank assembly accession: GCA_012979215.1
Helicoverpa armigera	337	1000.4	36.1	13,823	98.6	PRJNA378437<br>GenBank assembly accession: GCF_002156985.1
Bombyx mori	482	4008.4	37.7	13,850	95.8	PRJDA20217<br>GenBank assembly accession: GCF_000151625.1
Manduca sexta	384	755.8	35.3	15,451	93.1	PRJNA81037<br>GenBank assembly accession: GCF_000262585.1
Operophtera brumata	638	65.6	38.6	16,912	79.4	PRJNA263715<br>GenBank assembly accession: GCA_001266575.1
Danaus plexippus	249	715.7	31.6	15,232	95.3	PRJNA72423<br>GenBank assembly accession: GCA_000235995.2
Heliconius melpomene	275	2102.7	32.8	20,102	98.4	http://download.lepbase.org/
Plutella xylostella	393	737.2	38.3	18,106	91.6	PRJNA78271<br>GenBank assembly accession: GCF_000330985.1
Drosophila melanogaster	144	25286.9	42	13,916	99.6	PRJNA13812<br>GenBank assembly accession: GCF_000001215.4

Supplementary Table 8. Genes related to pesticide resistance and Bt resistance in fall armyworm.

Gene	Gene Number	Gene Length (bp)	CDS Length (bp)	CDS SNPs	CDS SNP Ratio	Pi
CYP450 (cytochrome p450)	FAW_001434	2613	1479	136	0.092	0.0104
AChE (acetylcholinesterase)	FAW_001505	5190	2117	108	0.051	0.0117
ABCC2	FAW_001807	13583	4047	425	0.105	0.0048
ABCC3	FAW_001808	24944	4080	362	0.089	0.0050
ABCB1	FAW_003193	14600	3870	180	0.047	0.0023
ABCA2	FAW_007144	14246	5238	383	0.073	0.0048
ABCG1	FAW_008619	19821	1824	155	0.085	0.0006
TSPAN (tetraspanin 1)	FAW_014576	11020	1056	108	0.102	0.0012
VGSC (voltage-gated sodium channel)	FAW_014653	28771	6165	234	0.038	0.0007
Cadherin	FAW_016242	21889	5073	254	0.050	0.0020
ALP1 (alkaline phosphatase 1)	FAW_016659	5017	3258	52	0.016	0.0009
APN1 (aminopeptidase N1)	FAW_017168	14962	3054	165	0.054	0.0025
RyR (ryanodine receptor)	FAW_017918	105101	15951	1510	0.095	0.0016
SR-C (scavenger receptor-C)	FAW_018001	6458	1572	224	0.142	0.0040

Supplementary Table 9. Distribution of mitochondrial insertion in 107 re-sequenced fall armyworm samples and American corn-strain (ASW) and rice-strain (AXE).

	Genome			Mitochondrial			Tpi			Insertion
Sample No.	Corn	Rice	heterozygous	Corn	Rice	heterozygous	Corn	Rice	heterozygous	Reads Number
ASW	1.000	0.000	0.000	1.000	0.000	0.000	1.000	0.000	0.000	0
AXE	0.000	1.000	0.000	0.000	1.000	0.000	0.000	1.000	0.000	0
AFR4	0.749	0.134	0.118	0.981	0.019	0.000	0.364	0.455	0.182	0
AFR5	0.748	0.135	0.118	0.981	0.019	0.000	0.318	0.500	0.182	0
AFR14	0.737	0.151	0.112	0.986	0.014	0.000	0.773	0.182	0.045	0
AFR15	0.739	0.145	0.117	0.986	0.014	0.000	0.727	0.136	0.136	0
YKM1	0.733	0.155	0.112	0.024	0.971	0.005	0.773	0.182	0.045	0
YKM2	0.728	0.157	0.115	0.981	0.019	0.000	0.773	0.136	0.091	1
GHZ1-1	0.731	0.153	0.116	0.024	0.967	0.010	0.727	0.182	0.091	8
GHZ2	0.728	0.156	0.116	0.024	0.962	0.014	0.773	0.182	0.045	27
GBL1	0.733	0.154	0.113	0.024	0.967	0.010	0.773	0.182	0.045	0
GLZ1	0.726	0.156	0.118	0.024	0.962	0.014	0.773	0.182	0.045	0
GLZ3	0.723	0.158	0.118	0.024	0.928	0.048	0.773	0.182	0.045	0
HY1	0.736	0.152	0.112	0.024	0.900	0.077	0.773	0.182	0.045	22
HY2	0.734	0.153	0.113	0.024	0.967	0.010	0.727	0.136	0.136	0
FS1	0.733	0.154	0.113	0.024	0.967	0.010	0.773	0.136	0.091	0
FS2	0.729	0.155	0.116	0.024	0.967	0.010	0.773	0.182	0.045	0
XW1	0.732	0.156	0.113	0.024	0.967	0.010	0.727	0.273	0.000	0
XW2	0.731	0.156	0.113	0.024	0.919	0.057	0.773	0.182	0.045	0

DG1	0.735	0.150	0.115	0.024	0.971	0.005	0.727	0.136	0.136	0
DG2	0.730	0.157	0.113	0.024	0.957	0.019	0.818	0.136	0.045	0
HZ1	0.729	0.153	0.117	0.024	0.971	0.005	0.773	0.182	0.045	0
HZ2	0.723	0.160	0.117	0.024	0.967	0.010	0.773	0.136	0.091	0
ZH1	0.729	0.157	0.114	0.024	0.967	0.010	0.773	0.182	0.045	0
ZH2	0.727	0.155	0.117	0.024	0.967	0.010	0.727	0.136	0.136	0
WC1	0.727	0.157	0.116	0.024	0.967	0.010	0.818	0.136	0.045	0
WC2	0.725	0.161	0.114	0.024	0.971	0.005	0.773	0.136	0.091	0
YF1	0.734	0.153	0.113	0.024	0.967	0.010	0.773	0.182	0.045	0
YF2	0.731	0.153	0.116	0.024	0.971	0.005	0.773	0.182	0.045	0
SG1	0.741	0.152	0.107	0.024	0.967	0.010	0.727	0.136	0.136	6
SG2	0.730	0.155	0.115	0.024	0.967	0.010	0.773	0.182	0.045	8
GHZ2-2	0.730	0.157	0.113	0.024	0.967	0.010	1.000	0.000	0.000	6
GWY1	0.731	0.152	0.117	0.986	0.014	0.000	0.773	0.182	0.045	0
GWY2	0.727	0.157	0.116	0.024	0.967	0.010	0.773	0.182	0.045	0
GNS1	0.723	0.157	0.120	0.024	0.967	0.010	0.773	0.000	0.227	0
GNS2	0.731	0.153	0.116	0.024	0.967	0.010	0.682	0.136	0.182	0
GQY1	0.729	0.155	0.116	0.024	0.967	0.010	0.773	0.182	0.045	2
GQY2	0.730	0.155	0.115	0.024	0.967	0.010	0.773	0.182	0.045	0
GLD1	0.728	0.156	0.116	0.024	0.971	0.005	0.727	0.136	0.136	2
GFS1	0.733	0.155	0.112	0.024	0.967	0.010	0.773	0.182	0.045	0
GFS2	0.727	0.157	0.117	0.024	0.967	0.010	0.773	0.000	0.227	0
GJX11	0.726	0.158	0.115	0.024	0.967	0.010	0.773	0.182	0.045	0
GJX12	0.722	0.160	0.118	0.024	0.967	0.010	1.000	0.000	0.000	0
GJX13	0.726	0.158	0.117	0.024	0.967	0.010	1.000	0.000	0.000	0

GJX15	0.731	0.155	0.115	0.024	0.967	0.010	0.727	0.136	0.136	0
GJX16	0.731	0.155	0.114	0.024	0.967	0.010	0.727	0.136	0.136	30
YZ1	0.730	0.157	0.113	0.024	0.967	0.010	0.773	0.182	0.045	0
YZ2	0.732	0.154	0.114	0.029	0.962	0.010	0.773	0.182	0.045	0
CZ1	0.732	0.153	0.115	0.024	0.967	0.010	0.773	0.182	0.045	12
CZ2	0.735	0.150	0.115	0.024	0.971	0.005	0.773	0.182	0.045	28
XP1	0.728	0.155	0.117	0.024	0.967	0.010	0.818	0.136	0.045	9
XP2	0.733	0.154	0.113	0.024	0.971	0.005	0.773	0.182	0.045	0
HSY1-BF	0.726	0.157	0.117	0.024	0.967	0.010	0.773	0.182	0.045	0
HN1	0.723	0.158	0.119	0.024	0.967	0.010	0.773	0.136	0.091	0
HSY1	0.735	0.151	0.114	0.024	0.971	0.005	0.773	0.000	0.227	6
HSY2	0.734	0.152	0.114	0.024	0.967	0.010	0.773	0.000	0.227	0
ZLS2	0.732	0.154	0.114	0.024	0.957	0.019	0.727	0.045	0.227	0
ZLQ1	0.731	0.153	0.115	0.024	0.957	0.019	0.727	0.136	0.136	0
SHZ1	0.729	0.158	0.113	0.024	0.967	0.010	0.773	0.182	0.045	2
SXX1	0.733	0.152	0.115	0.024	0.971	0.005	0.682	0.136	0.182	0
JJA1	0.729	0.154	0.117	0.024	0.967	0.010	0.773	0.182	0.045	38
JJA2	0.730	0.155	0.116	0.024	0.967	0.010	0.773	0.182	0.045	17
JPX1	0.733	0.144	0.123	0.024	0.967	0.010	0.773	0.000	0.227	3
JPX2	0.729	0.156	0.115	0.024	0.967	0.010	1.000	0.000	0.000	3
JHK1	0.727	0.154	0.119	0.024	0.952	0.024	0.773	0.182	0.045	0
JJJ1	0.724	0.160	0.116	0.024	0.971	0.005	0.773	0.182	0.045	0
JJJ2	0.731	0.154	0.115	0.024	0.971	0.005	0.773	0.182	0.045	15
JJJ3	0.729	0.155	0.116	0.024	0.971	0.005	0.773	0.182	0.045	0
JJJ4	0.724	0.157	0.119	0.024	0.967	0.010	0.773	0.182	0.045	48

JNC1	0.727	0.157	0.116	0.024	0.971	0.005	0.818	0.136	0.045	0
JNC2	0.728	0.155	0.117	0.024	0.967	0.010	0.773	0.000	0.227	0
JNC3	0.728	0.156	0.116	0.024	0.967	0.010	0.773	0.182	0.045	0
JNC4	0.727	0.154	0.119	0.024	0.967	0.010	0.773	0.136	0.091	0
JXY1	0.728	0.154	0.118	0.024	0.971	0.005	0.773	0.182	0.045	4
JXY2	0.726	0.158	0.117	0.024	0.967	0.010	0.773	0.182	0.045	0
JJJ11	0.726	0.156	0.118	0.024	0.967	0.010	0.818	0.136	0.045	24
JJJ12	0.741	0.144	0.114	0.024	0.962	0.014	0.773	0.136	0.091	0
JNC12	0.731	0.154	0.115	0.024	0.962	0.014	0.773	0.136	0.091	0
SFX1	0.728	0.154	0.118	0.024	0.962	0.014	0.773	0.182	0.045	0
HJZ2	0.732	0.153	0.115	0.024	0.967	0.010	0.773	0.182	0.045	3
HXG1	0.732	0.154	0.114	0.024	0.967	0.010	0.773	0.182	0.045	15
HXG2	0.727	0.155	0.118	0.024	0.967	0.010	0.727	0.136	0.136	0
HTS1	0.728	0.155	0.117	0.029	0.962	0.010	1.000	0.000	0.000	0
HTS2	0.725	0.155	0.120	0.033	0.957	0.010	0.773	0.000	0.227	0
HYC1	0.728	0.155	0.117	0.024	0.967	0.010	0.773	0.182	0.045	0
HYC2	0.724	0.155	0.121	0.024	0.967	0.010	0.773	0.136	0.091	0
HXZ1	0.732	0.153	0.115	0.024	0.971	0.005	0.773	0.182	0.045	0
HXZ2	0.728	0.155	0.117	0.024	0.967	0.010	0.773	0.182	0.045	0
CWX1	0.730	0.156	0.113	0.024	0.967	0.010	0.773	0.182	0.045	20
CWX2	0.727	0.154	0.118	0.024	0.971	0.005	0.773	0.136	0.091	19
CXS1	0.733	0.152	0.115	0.024	0.967	0.010	0.818	0.136	0.045	0
CXS2	0.729	0.155	0.115	0.024	0.962	0.014	0.773	0.182	0.045	0
CQJ2	0.728	0.156	0.117	0.024	0.962	0.014	0.818	0.136	0.045	0
XLZ1	0.730	0.155	0.116	0.986	0.014	0.000	0.773	0.182	0.045	0

AWJ1	0.734	0.153	0.113	0.024	0.967	0.010	0.773	0.182	0.045	0
AWJ2	0.726	0.155	0.119	0.986	0.014	0.000	0.773	0.000	0.227	0
AWH1	0.724	0.158	0.118	0.024	0.971	0.005	1.000	0.000	0.000	0
AWH2	0.729	0.155	0.116	0.024	0.976	0.000	0.773	0.000	0.227	0
AAQ1	0.729	0.156	0.115	0.024	0.971	0.005	1.000	0.000	0.000	0
AAQ2	0.726	0.155	0.119	0.024	0.967	0.010	0.773	0.136	0.091	0
ACZ1	0.729	0.156	0.115	0.024	0.967	0.010	0.773	0.182	0.045	19
ACZ2	0.732	0.155	0.113	0.024	0.967	0.010	0.773	0.182	0.045	12
ACZ3	0.727	0.154	0.119	0.024	0.971	0.005	0.773	0.182	0.045	0
ACZ4	0.730	0.155	0.115	0.024	0.967	0.010	0.773	0.182	0.045	0
JHM1	0.731	0.156	0.113	0.024	0.967	0.010	0.727	0.136	0.136	0
GGY1	0.726	0.156	0.118	0.024	0.967	0.010	0.727	0.136	0.136	0
GGY2	0.731	0.154	0.115	0.024	0.967	0.010	0.773	0.000	0.227	0
SLS1	0.732	0.155	0.114	0.024	0.967	0.010	0.727	0.136	0.136	0
SLS2	0.726	0.157	0.117	0.024	0.967	0.010	0.773	0.182	0.045	0

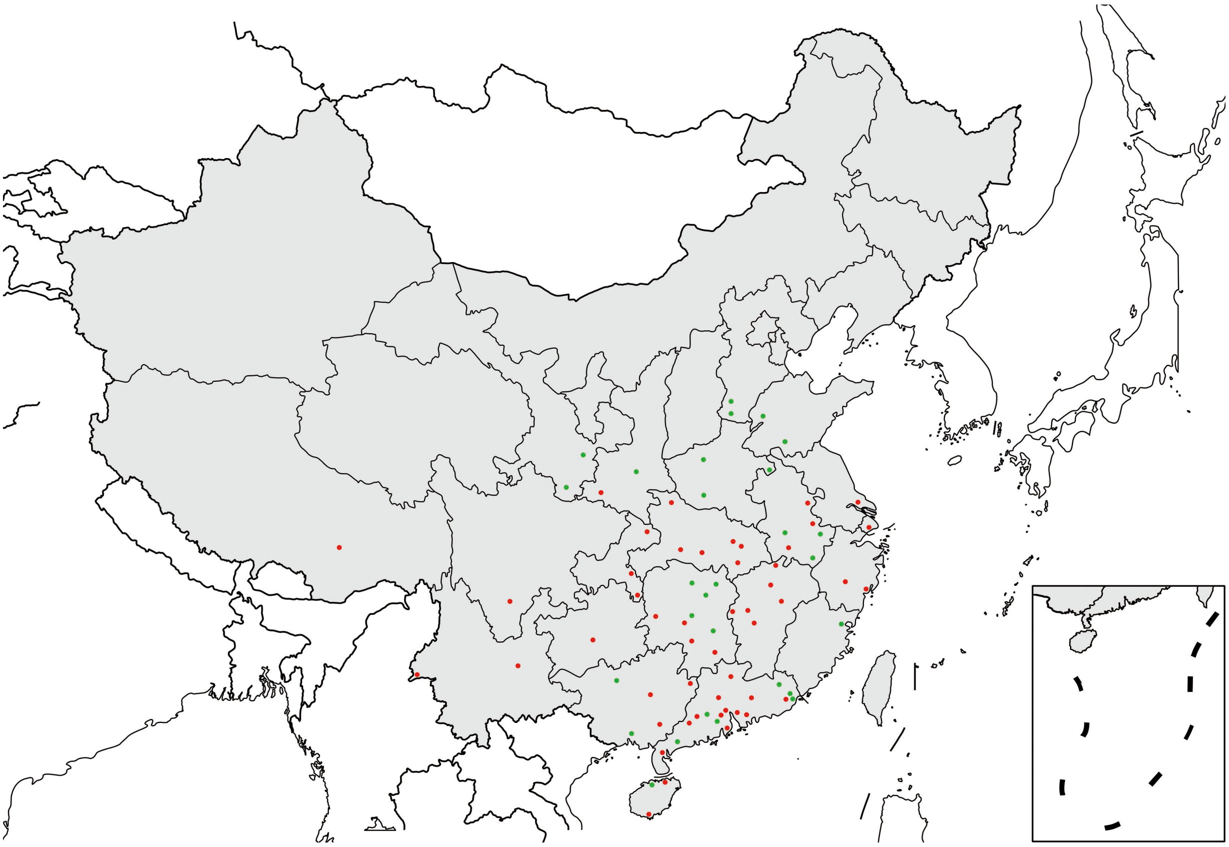

Supplementary Figure 1 Map of fall armyworm collection sites in China. Each point represents a city, the red point represents the source of the re-sequenced samples from 16 Provinces in China, the green points represents the source of some rest samples from other 5 Provinces used for population genetic analysis in this study.

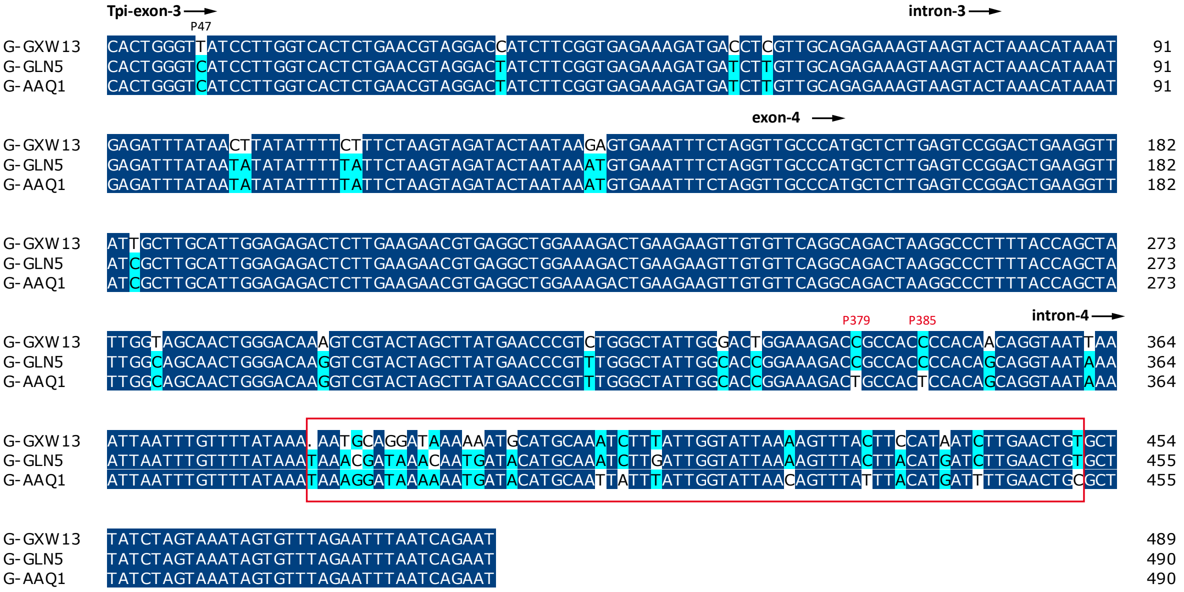

Supplementary Figure 2 Comparison of different Tpi genotypes in Chinese samples. G-GXW13 represents Africa-specific genotype, G-GLN5 and G-AAQ1 represent C-strain genotype with difference in intron4 region. Red box represents the sequence variation in intron4 region linked to two variable sites (P379/385) in exon-4 of Tpi gene.
